# A recent overview on the synthesis of 1,4,5-trisubstituted 1,2,3-triazoles

**DOI:** 10.3762/bjoc.17.114

**Published:** 2021-07-13

**Authors:** Pezhman Shiri, Ali Mohammad Amani, Thomas Mayer-Gall

**Affiliations:** 1 Department of Medical Nanotechnology, School of Advanced Medical Sciences and Technologies, Shiraz University of Medical Sciences, Shiraz, Iran; 2 Pharmaceutical Sciences Research Center, Shiraz University of Medical Sciences, Shiraz, Iran; 3 Department of Physical Chemistry and Center of Nanointegration (CENIDE), University of Duisburg-Essen, Universitätsstr. 2, 45141 Essen, Germany; 4 Deutsches Textilforschungszentrum Nord-West gGmbH, Adlerstr. 1, 47798 Krefeld, Germany

**Keywords:** azides, Click reaction, [3 + 2]‐cycloaddition, fully functionalized 1,2,3-triazoles, N-containing heterocycles, 1,4,5-trisubstituted 1,2,3-triazoles

## Abstract

Diverse strategies for the efficient and attractive synthesis of a wide variety of relevant 1,4,5-trisubstituted 1,2,3-triazole molecules are reported. The synthesis of this category of diverse fully functionalized 1,2,3-triazoles has become a necessary and unique research subject in modern synthetic organic key transformations in academia, pharmacy, and industry. The current review aims to cover a wide literature survey of numerous synthetic strategies. Recent reports (2017–2021) in the field of 1,4,5-trisubstituted 1,2,3-triazoles are emphasized in this current review.

## Introduction

**Figure 1:**
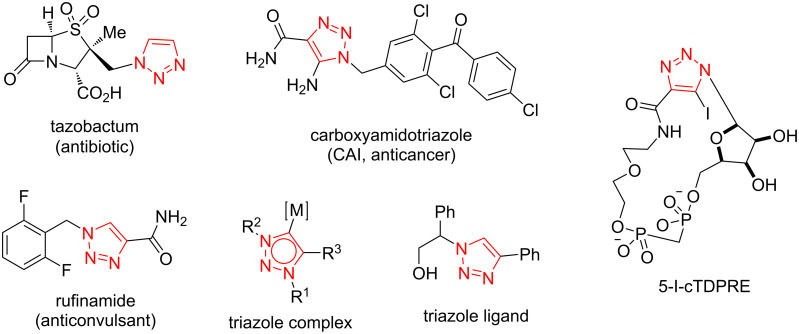
Some significant triazole derivatives [8,23–27].

A high number of N-heterocycles [1–4] are identified, and this number is increasing very quickly [5–8]. Among them, the small heterocyclic ring of the 1,2,3-triazole is present in a broad variety of compounds with not only biological but also industrial significance [9–11]. It possesses a cyclic scaffold with carbon and three nitrogen elements in the ring [12–15]. An immense versatility of biological properties is possessed by 1,2,3‐triazole heterocyclic systems, and many strategies are screened for the synthesis of these rings [16–17]. Notably, triazole rings exhibit various medicinal applications, such as usages as anticancer [18] anti-HIV [19], antimalarial [20], antiplasmodial [21], and antibacterial agents [1,22]. Some significant triazole derivatives in this context are shown in Figure 1 [8,23–27].

As such, there is still a demand for the expansion of ways to access new 1,2,3-triazole heterocyclic rings in rapid, efficient, and versatile ways. Triazole rings could serve as important intermediates for numerous important organic transformations [28–30]. Diverse applications of these five-membered N-heterocycles are the result of both their easy synthetic procedures and ring functionalization [9,31–32]. Typically, 1,2,3-triazole derivatives are prepared via a 1,3-dipolar cycloaddition reaction between azides and alkynes [33]. It is worth noting that the metal-catalyzed Huisgen cycloaddition reaction could provide disubstituted 1,2,3-triazoles. The selective introduction of substituents to three different positions on the 1,2,3-triazole frame can notably improve the features of the molecule. Therefore, practical and cost-effective strategies for the selective preparation of 1,4,5-trisubstituted 1,2,3-triazoles have gained much attention in triazole chemistry. Generally, two protocols for the synthesis of this type of triazole derivatives could be mentioned (metal or metal-free catalysis).

Contrary to sporadic attempts [34], there is no detailed, systematic, and updated overview covering the synthesis and mechanism aspects of 1,4,5-trisubstituted 1,2,3-triazoles. It is worth noting that several elegant reviews on the subject of substituted triazoles have been published in the last decade [34–36]. A valuable overview on earlier works on the subject of the cyclization reactions between alkynes and nitrogen sources for the synthesis of triazoles has been presented by Zeni and Neto. This review focuses on the preparation of disubstituted triazoles. However, the synthetic routes of a few 1,4,5-trisubstituted 1,2,3-triazoles have been displayed in this review [34]. In 2010, the 3,4,5-trisubstituted 1,2,4-triazoles have also been surveyed in a significant review by Fehrentz et al. This review mainly focuses on the preparation of monocyclic 3,4,5-trisubstituted 1,2,4-triazoles [37]. In 2017, different methods for the preparation of a range of 1,4,5-trisubstituted 1,2,3-triazoles through multicomponent reactions and the relevant mechanistic aspects have been surveyed by Chen, Ren, et al. [27].

**Scheme 1:**
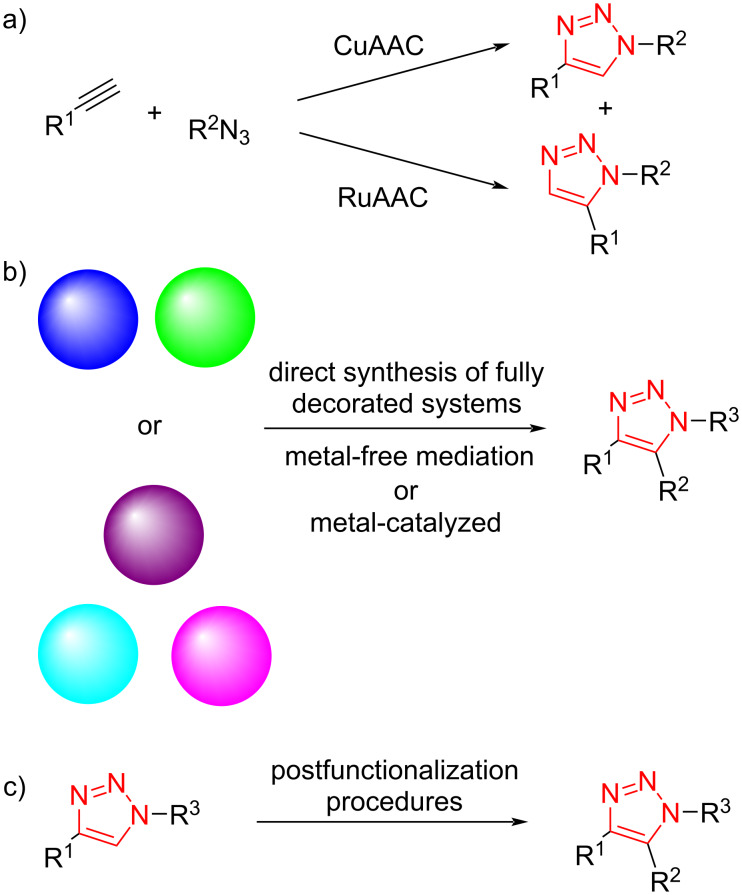
A general comparison between synthetic routes for disubstituted 1,2,3-triazole derivatives and fully substituted 1,2,3-triazole derivatives.

The 1,3-dipolar cycloaddition reaction between azides and alkynes is the most efficient pathway for the preparation of disubstituted 1,2,3-triazole derivatives. The copper-catalyzed azide–alkyne cycloaddition (CuAAC) for the synthesis of 1,4-disubstituted 1,2,3-triazole derivatives was initially discovered by the groups of Meldal and Sharpless. Then, Ru-catalyzed azide–alkyne cycloaddition (RuAAC), affording selectively 1,5-disubstituted 1,2,3-triazoles, was introduced [38]. In recent years, fully substituted 1,2,3-triazole derivatives have been studied by many research groups (Scheme 1). Among an extensive range of 1,2,3-triazoles, fully decorated 1,2,3-triazoles display a sort of outstanding substance found in plenty biologically important molecules and functionalized materials. As such, the discovery of more effective strategies for the rapid preparation of these significant products is extremely desired. The regioselective synthesis of these compounds mainly includes (i) direct synthesis of fully decorated systems and (ii) postfunctionalization of disubstituted 1,2,3-triazoles. The postfunctionalization of disubstituted 1,2,3-triazole was also explained with a range of C–C- and C–N-bond-forming reactions. A number of fully substituted 1,2,3-triazoles was provided from different compounds in moderate to high yield under mild conditions. In the last years, the exploitation of triazole rings as beneficial directing groups to produce complex triazoles has been screened. Although the postfunctionalization of disubstituted 1,2,3-triazoles may include multistep reactions for the synthesis of this kind of products, it is an easy and efficient strategy (Scheme 1).

The current review considers the synthesis of diverse 1,4,5-trisubstituted 1,2,3-triazoles via metal- and metal-free catalytic routes. Synthesis and mechanism aspects of this category of fully functionalized triazoles are reported in detail in the current review article. We believe a precise and systematic overview on the established protocols for the synthesis of diverse fully decorated triazoles would be helpful to a wide community of researchers working in chemistry laboratories and industries. The present review is intended to describe new reports (2017 to present) concerning the synthesis of diverse fully decorated triazoles. As shown in Figure 1, 1,4,5-trisubstituted 1,2,3-triazole derivatives display a privileged class of fully decorated triazoles in Click chemistry extensively found in many biologically important compounds and functionalized frameworks. In this regard, the discovery of diverse procedures for the efficient preparation of these significant products is highly required.

## Review

### Metal-free synthesis of fully decorated triazoles

**Scheme 2:**
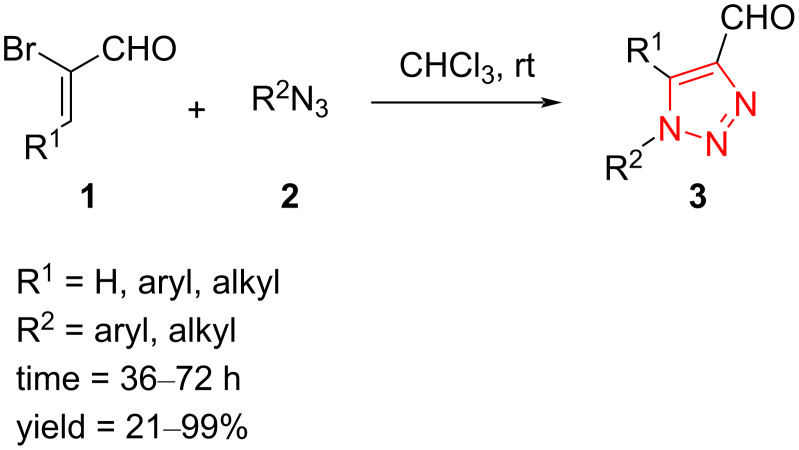
Synthesis of formyltriazoles **3** from the treatment of α-bromoacroleins **1** with azides **2**.

Developing metal-free protocols for the synthesis of triazole derivatives has been considered as growingly attractive yet challenging objectives in the environmental catalysis area. A metal-free protocol has been developed for the synthesis of 1,4,5-trisubstituted 1,2,3-triazoles **3** from the reaction between α-bromo-β-alkylacroleins and α-bromo-β-phenylacroleins **1** and organic azides **2** in chloroform at room temperature (Scheme 2). A low to moderate yield of products was achieved using β-methyl-substituted, long-chain heptyl-substituted, and sterically hindered cyclohexyl-substituted azides. The α-bromo-β-phenylacroleins bearing electron-donating or electron-withdrawing substituents produced the corresponding trisubstituted **3** in good to moderate yield irrespective of the nature and position of the substituent [39].

**Scheme 3:**
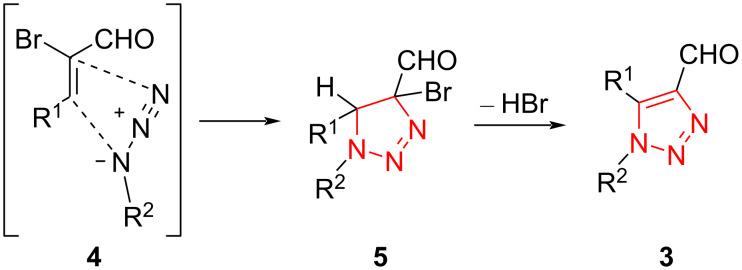
A probable mechanism for the synthesis of formyltriazoles **5** from the treatment of α-bromoacroleins **1** with azides **2**.

The supposed reaction mechanism for the reaction is shown in Scheme 3. Initially, the presence of bromine as an electron-withdrawing substituent lowers the LUMO energy to facilitate the cycloaddition process of acrolein with organic azide. The subsequent elimination of HBr leads to the triazole ring [39].

**Scheme 4:**
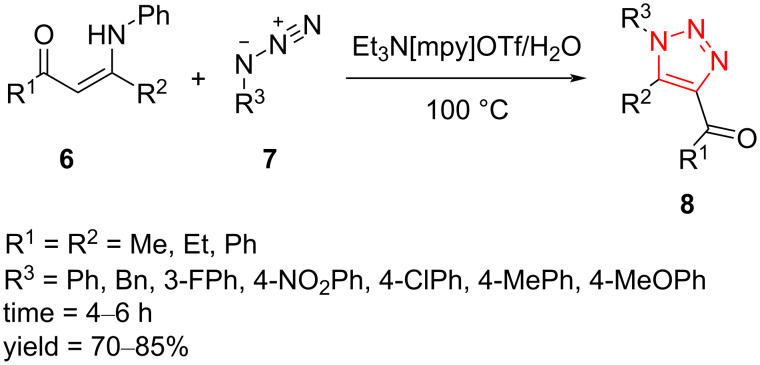
Synthesis of 1,4,5-trisubstituted 1,2,3-triazoles **8** from the reaction of aryl azides **7** with enaminones **6**.

Another metal-free strategy to 1,4,5-trisubstituted 1,2,3-triazoles **8** was realized by regioselective reaction of aryl azides **7** with enaminones **6** in the presence of triethylamine, water, and ionic liquid (IL). This method exhibits good functional group compatibility, including using enaminones containing aliphatic and aromatic substituents as well as azide compounds containing electron-donating and -withdrawing groups on the aromatic ring. In all cases, only 4-acyl-substituted regioisomers were obtained (Scheme 4) [40].

**Scheme 5:**
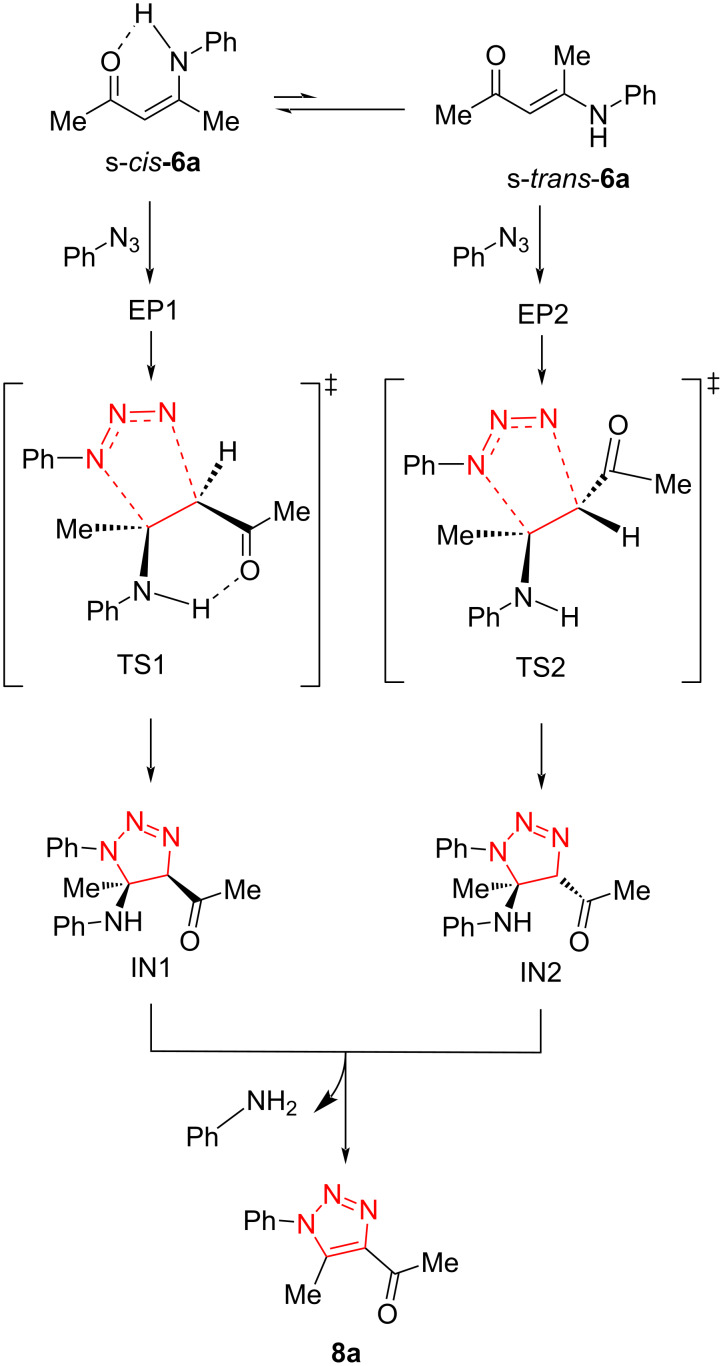
Proposed mechanism for the synthesis of 1,4,5-trisubstituted 1,2,3-triazoles from the reaction of aryl azides with enaminones.

The mechanism was also investigated by means of DFT calculations using the reaction between enaminone **6a** and phenyl azide. TS1 and TS2 have been proposed as two transition states, which then converted to IN1 and IN2 as two possible isomers. The stable final products were achieved via a cascade reaction including the elimination of aniline (Scheme 5) [40].

**Scheme 6:**
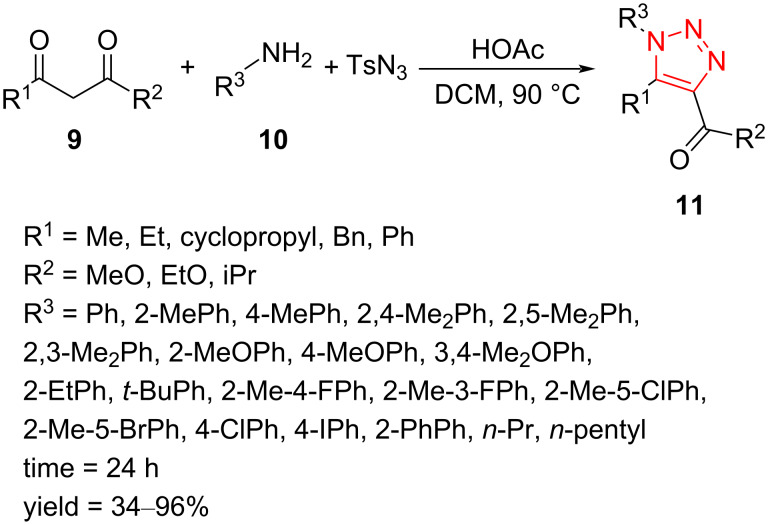
Synthesis of 1,4,5-trisubstituted 1,2,3-triazoles **11** from the reaction of primary amines **10** with 1,3-dicarbonyl compounds **9**.

A one-pot and multicomponent route to 1,4,5-trisubstituted 1,2,3-triazoles **11** containing a carboxylic ester group on the triazole ring was reported by Zhao et al. This strategy generates desired products from the reaction of readily available primary amines **10**, 1,3-dicarbonyl compounds **9**, and tosyl azide through a cycloaddition process in DCM as solvent and HOAc as additive at 90 °C in moderate to excellent yield (Scheme 6) [41].

**Scheme 7:**
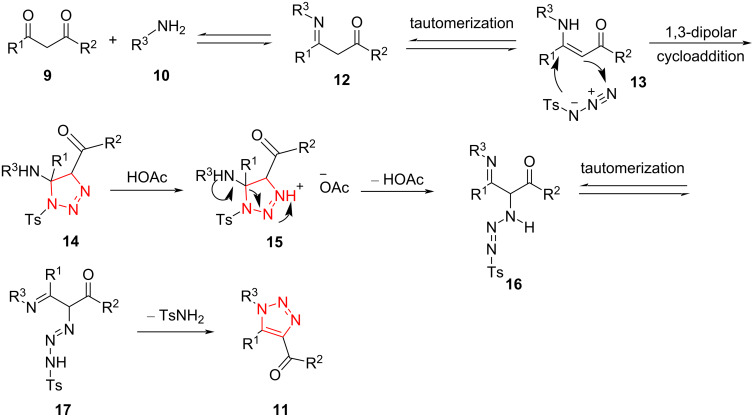
The proposed mechanism for the synthesis of 1,4,5-trisubstituted 1,2,3-triazoles **11** from the reaction of primary amines **10** with 1,3-dicarbonyl compounds **9**.

As illustrated in Scheme 7, imine intermediate **12** was generated via the reaction between amine **10** and 1,3-dicarbonyl compound **9**. Then, tautomerization of the imine intermediate produces the intermediate enamine **13**. The 1,3-dipolar cycloaddition of enamine **13** with azide produces intermediate 1,2,3-triazoline **14**. In continuation, a ring-opening process is promoted by acetic acid to afford the intermediate **16**. Afterward, triazene intermediate **17** was achieved by a tautomerization process. A highly decorated 1,2,3-triazole **11** was formed via cyclization of the intermediate **17** along with the loss of one molecule of TsNH_2_ [41].

**Scheme 8:**
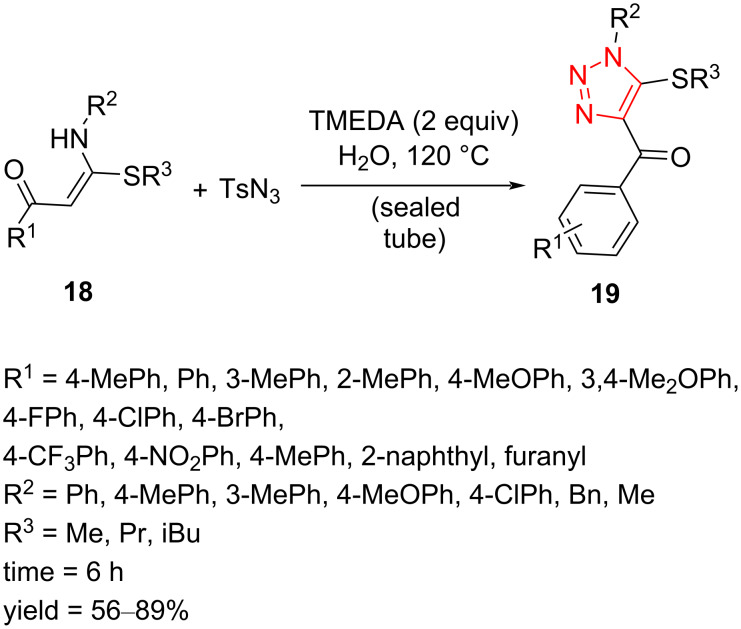
Synthesis of fully decorated 1,2,3-triazoles **19** containing a sulfur-based side chain.

Fully decorated 1,2,3-triazoles **19** containing a sulfur-based side chain have been synthesized via a cyclization of β-thiolated enaminones **18** with tosyl azide. A wide range of desired 5-thiolated 1,2,3-triazoles **19** has been prepared in good to high yield in H_2_O as solvent using TMEDA as a weak base, thus providing an environmentally friendly procedure that was demonstrated to be practicable also on a gram scale. A wide range of β-thiolated arylenaminones **19** containing electron-donating and -withdrawing groups were treated with azide under the standard reaction conditions. A variety of groups R^1^ in the phenyl ring, such as Me, MeO, CF_3_, Br, Cl, F, NO_2_, Et, Pr, and iBu were tolerated under the reaction conditions to afford the desired fully substituted triazoles in good to high yield. The phenyl ring could also be replaced by naphthyl and furyl rings to give the corresponding products in good to excellent yield. Besides, aryl and alkyl groups for R^2^ displayed applicability in the preparation of corresponding 1,4,5-trisubstituted 1,2,3-triazoles. However, *N*-aryl products were yielded with slightly higher yield than the N-alkylated ones. Furthermore, it is notable that different alkylthio-containing starting materials, such as Et-, Pr-, and iBu-substituted thiolated enaminones, could react with azide substrate to produce corresponding triazole products in moderate yield (Scheme 8) [42].

**Scheme 9:**
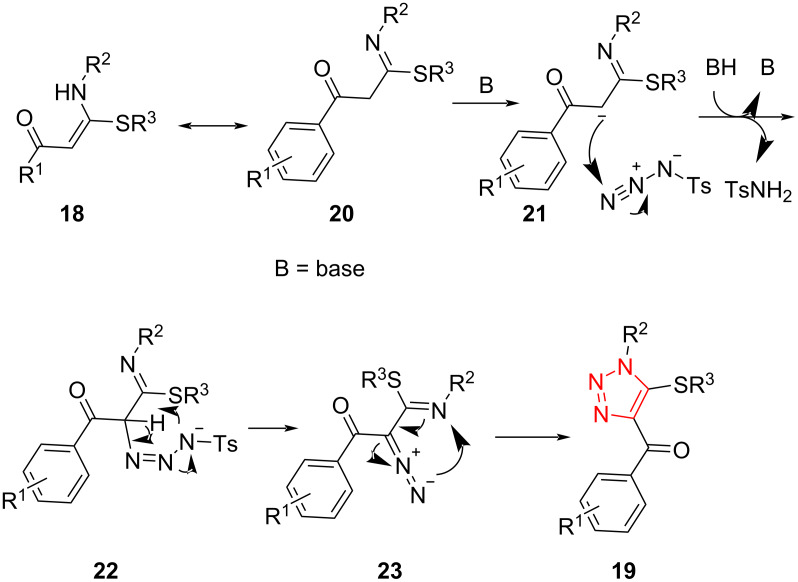
Mechanism for the formation of fully decorated 1,2,3-triazoles **19** containing a sulfur-based side chain.

The authors proposed a reaction mechanism in which the β-thioenaminone **18** tolerates deprotonation to afford anionic intermediate **21** through the tautomer **20** in the presence of a base. In the next step, the nucleophilic addition of **21** to the azide takes place to form intermediate **22**, which subsequently converts to diazo-functionalized intermediate **23**. Finally, the intramolecular annulation of intermediate **23** gives corresponding fully decorated 1,2,3-triazole **19** (Scheme 9) [42].

**Scheme 10:**
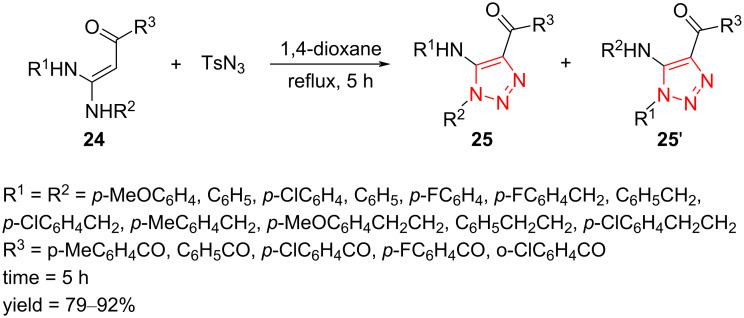
Synthesis of fully decorated 1,2,3-triazole compounds **25** through the regioselective addition and cyclization reaction of EDAMs **24** with *p*-methylbenzenesulfonyl azide.

A domino coupling synthesis of fully decorated 1,2,3-triazole compounds **25** through the regioselective addition and cyclization reaction of 1,1-enediamines (EDAMs) **24** with *p*-methylbenzenesulfonyl azide in 1,4-dioxane as solvent at reflux was presented by Yan et al. Some substituted EDAMs (R^1^ = R^2^) were concluded to be treated with TsN_3_. In continuation, substituted EDAMs (R^1^ ≠ R^2^) were also applied to check the regioselectiveness of this addition and cyclization reaction. The methodology provides a simple, straightforward, and facile path to access fully decorated 1,2,3-triazoles **25** in moderate to good yield. Noticeably, this uncatalyzed addition and cyclization process displayed good regioselectivity (Scheme 10) [23].

**Scheme 11:**
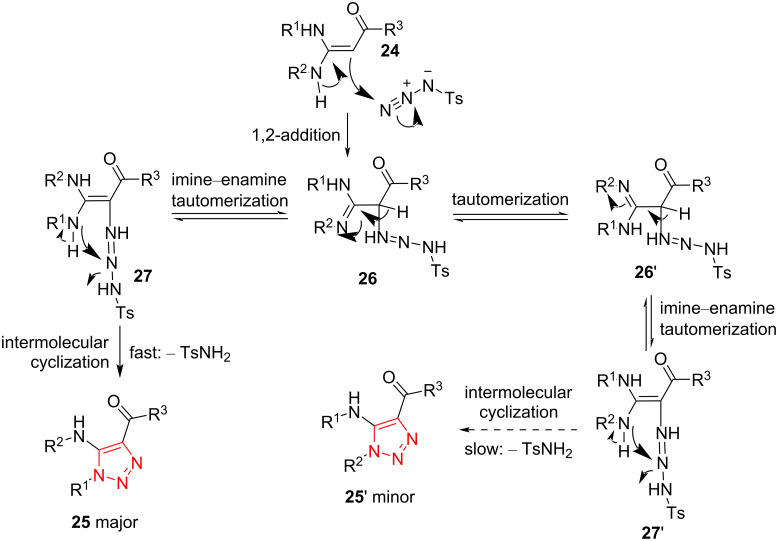
A reasonable mechanism for the synthesis of fully decorated 1,2,3-triazole compounds **25** through the regioselective addition and cyclization reaction of EDAMs **24** with *p*-methylbenzenesulfonyl azide.

The authors proposed that the reaction may proceed through the mechanism illustrated in Scheme 11. Initially, EDAM **24** is treated with an azide source and subsequently, the intermediate **26** is formed through 1,2-addition reaction. The intermediate **26** is imine–enamine-tautomerized to afford the intermediate **27**. Then, the final triazole compound, as major isomer, is achieved through an intermolecular cyclization process and elimination of one unit of TsNH_2_ [23].

**Scheme 12:**
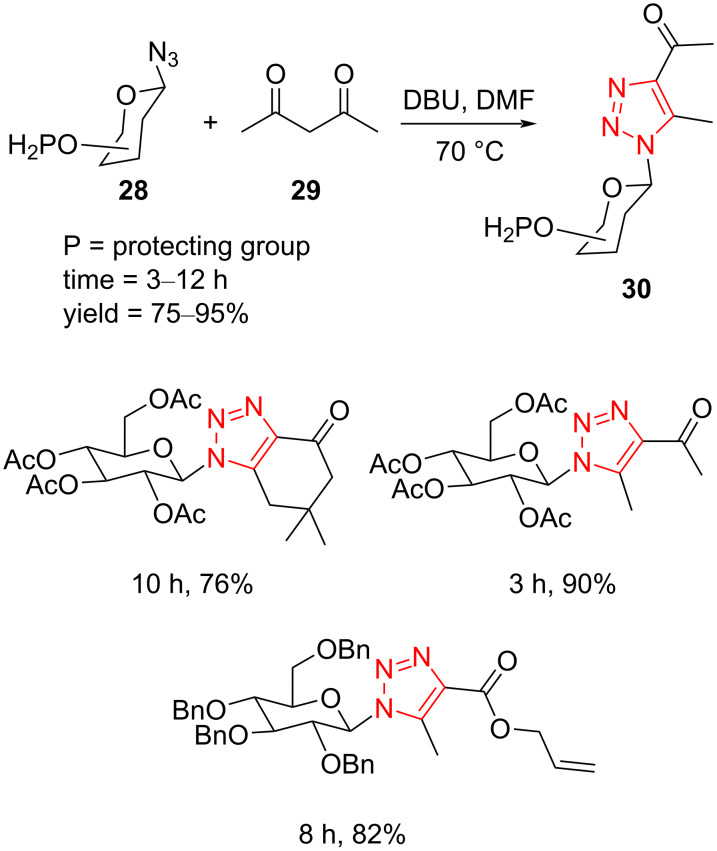
Synthesis of 1,4,5-trisubstituted glycosyl-containing 1,2,3-triazole derivatives **30** from the reaction of glycosyl azides **28** with enolates of active ketones and esters **29**.

A general strategy was described for the synthesis of 1,4,5-trisubstituted glycosyl-containing 1,2,3-triazole derivatives **30** from the reaction of glycosyl azides **28** with enolates of active ketones and esters **29** in the presence of DBU in DMF at 70 °C (Scheme 12) [43]. Diverse monosaccharide and disaccharide azides were successfully reacted with a variety of enolates of active ketones and esters **29**, such as 1,3-diketones, β-ketoesters, cyclic 1,3-diketone (dimedone), and β-ketoamide, respectively, to afford desired 1,4,5-trisubstituted 1,2,3-triazoles containing glycosyl with high yield. It should be pointed out that no byproducts were recognized under optimized reaction conditions. In addition, a range of functional groups have been investigated, and the results exhibited that they were unaffected. Finally, it would be possible to scale up the reaction with a negligible decrease in the yield of product [43].

**Scheme 13:**
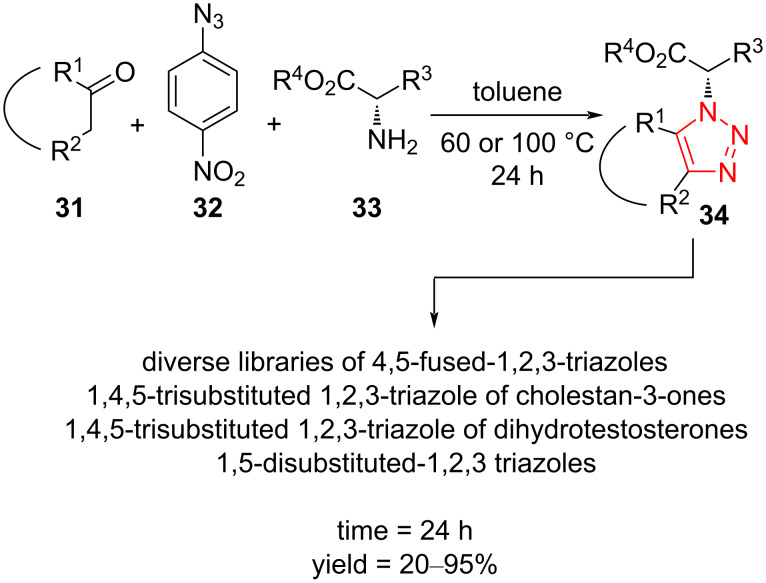
Synthesis of 1,4,5-trisubstituted 1,2,3-triazoles **34** via intramolecular cyclization reaction of ketones **31**, PNA (**32**), and amino esters **33**.

The synthesis of enantiomerically pure 1,4,5-trisubstituted 1,2,3-triazoles **34** via intramolecular cyclization reaction of ketones **31**, *p*-nitrophenyl azide (PNA, **32**) and amino esters **33** has been described by Dehaen et al. The products were often obtained in good yield and in all cases with the retention of the configuration of the stereocenter. The reaction was carried out by employing a variety of amino esters and a wide range of (hetero)aromatic and alkyl ketones. Natural products, such as 5*α*-cholestan-3-one and dihydrotestosterone, were also tolerated, affording a new strategy to modify biologically active compounds. Amino acids, such as tyrosine, ʟ-Phe-ʟ-Phe, glutamic acid, and serine derivatives were proceeded to extend the scope of the reaction (Scheme 13) [44].

**Scheme 14:**
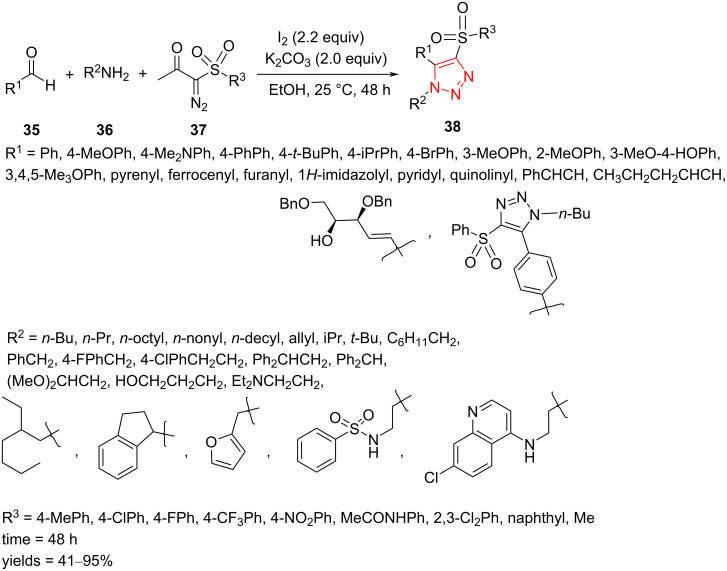
Synthesis of fully decorated 1,2,3-triazoles **38** from the reaction of aldehydes **35**, amines **36**, and α-diazo-β-ketosulfones **37**.

An efficient domino three-component route has been developed for the synthesis of fully decorated 1,2,3-triazoles **38** bearing a sulfonyl group in position 4. The synthesis required aldehydes **35**, amines **36**, α-diazo-ß-ketosulfones **37**, I_2_, and K_2_CO_3_ in EtOH. The scope and limitations of the reaction were assessed using alkyl aldehydes, (hetero)aryl aldehydes, amines, and diazosulfones. Reactions of aryl aldehydes containing a range of different functional groups gave products **38** in very good yield. Because of the faster hydrolysis of the in situ generated imine intermediates, aromatic aldehydes containing electron-withdrawing substituents afforded no product in the reaction. It was proved that a variety of aliphatic primary amines can efficiently produce the triazole products (Scheme 14) [45].

**Scheme 15:**
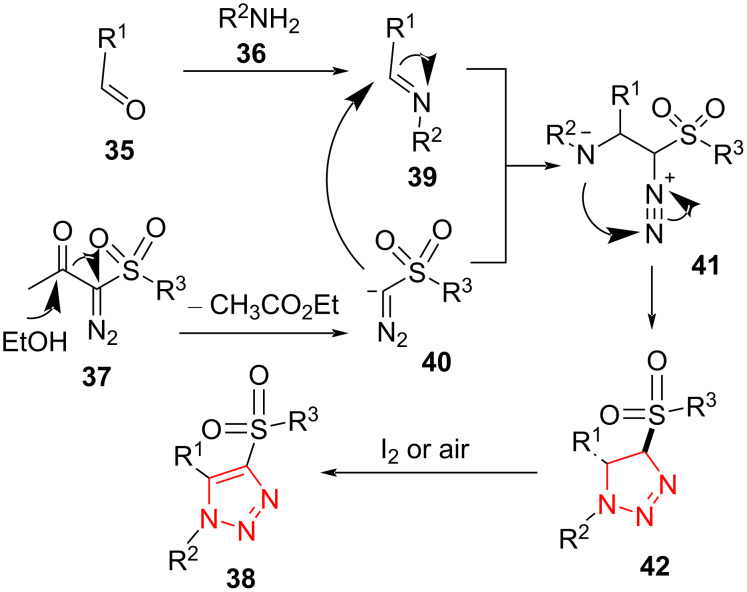
A reasonable mechanism for the synthesis of fully decorated 1,2,3-triazoles **38** from the reaction of aldehydes **35**, amines **36**, and α-diazo-β-ketosulfones **37**.

This one-pot tandem reaction proceeds via formation of a Schiff-base **39** and subsequently 1,3-dipolar cycloaddition to produce the intermediate **42**. Then, the intermediate **42** affords the final product **43** via an oxidative aromatization process in the presence of air or iodine (Scheme 15). To propose a precise mechanism, a cyclization reaction was carried out without molecular iodine in an inert atmosphere. No final product was observed, and only **42** was achieved in good yield. However, **42** was effectively transformed into the corresponding product with excellent yield in the presence of molecular iodine (Scheme 15) [45].

In 2020, Ramachary et al. reported the 1,3-dipolar cycloaddition of various enones **43** and **46** with less reactive vinyl/alkyl/aryl azides **44** via an enolate-mediated organocatalyst. This protocol provides diverse double C- and N-vinylated 1,2,3-triazole derivatives and C-vinylated 1,2,3-triazole derivatives from azidophilic substrates and different azide derivatives. A short reaction time, good to high yield of products, high diversity, high selectivity, and ease of operation were some benefits of this methodology. The enolate reactivity with azides was compared to enamines. The best conditions were the use of a catalytic amount of DBU in DMSO at room temperature [46].

**Scheme 16:**
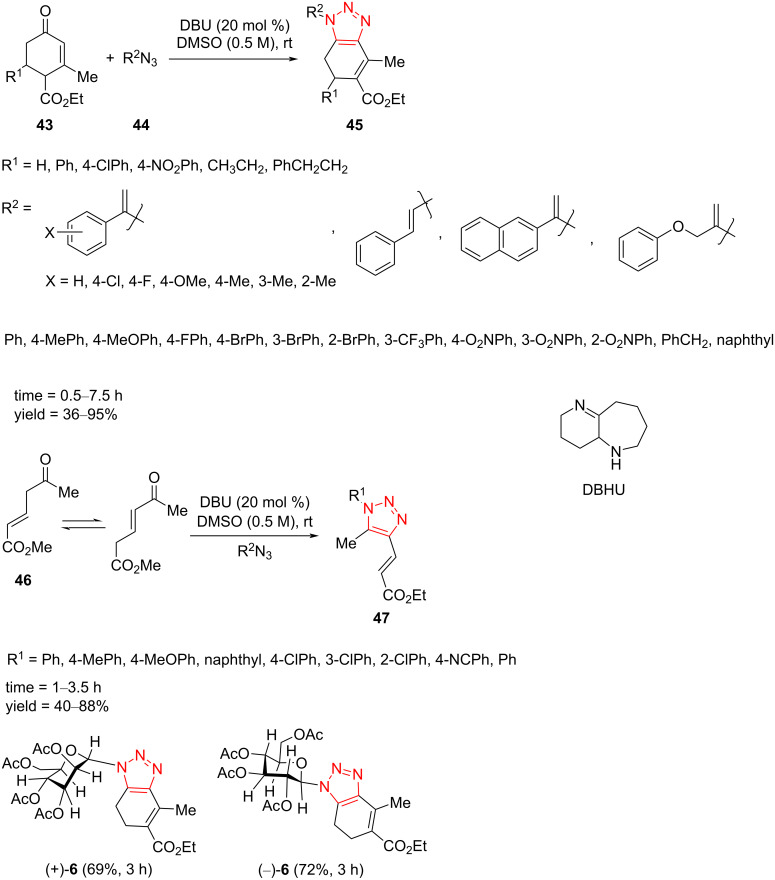
Synthesis of functionally rich double C- and N-vinylated 1,2,3-triazoles **45** and **47**.

The cyclic enones **43** were reacted with the α-azidostyrenes **44** containing groups such as Cl, F, and OMe to form the corresponding products (Scheme 16). The *o*-, *m-*, and *p*-tolylvinyl azides facilitated a good to excellent yield of the products. The tolylvinyl azide substituted with methyl at the *ortho* position afforded a lower yield of the desired product. The vinyl azides containing 2-naphthyl, β-phenyl, and 1-((2-azidoallyl)oxy)-4-nitrobenzenevinyl azides formed the corresponding products in high yield as well. Moreover, the alkyl-substituted cyclic enones treated successfully with azidophiles to give good yield of the corresponding double C- and N-vinylated 1,2,3-triazole derivatives **45**. Then, the reaction was extended to some aryl and alkyl azides and different cyclic enones. Moreover, a variety of vinyl, alkyl, and aryl azides were also applicable in the reaction. In continuation of the study, various alkyl- and aryl-substituted unmodified cyclic enones were explored, and the result was a moderate to good yield of the corresponding products. The reaction was performed well using alkyl-substituted cyclic enone. Finally, acyclic enone **46** reacted with aryl and vinyl azides to afford a moderate to high yield of the triazole products (Scheme 16) [46].

**Scheme 17:**
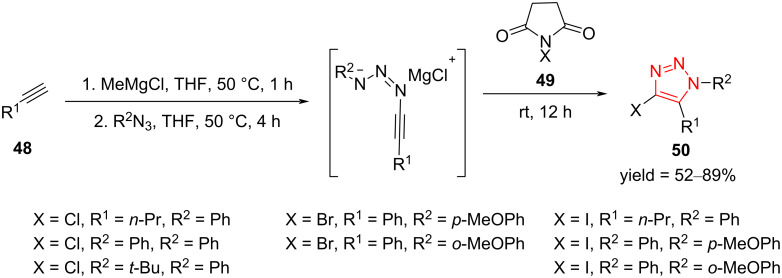
Synthesis of disubstituted 4-chloro-, 4-bromo-, and 4-iodo-1,2,3-triazoles **50**.

A simple one-pot two-stage strategy for the synthesis of disubstituted 4-chloro-, 4-bromo-, and 4-iodo-1,2,3-triazoles **50** from the reaction of the corresponding nonactivated alkynes **48** with organic azides was studied by Smirnov et al. The alkynes **48** were subjected to the action of MeMgCl in THF and then, the desired aryl azides were added into the solution, followed by the addition of *N*-halosuccinimide to give chloro-, bromo-, and iodotriazoles **50** (Scheme 17) [47]. It was found that the reaction requires a strict control of the temperature in the different steps, while the nature and position of the substituents of the aryl azide do not influence the yield of the reaction. The use of aliphatic alkynes, as well as the replacement of bromine and chlorine with iodine, leads to a decrease in the yield [47].

**Scheme 18:**
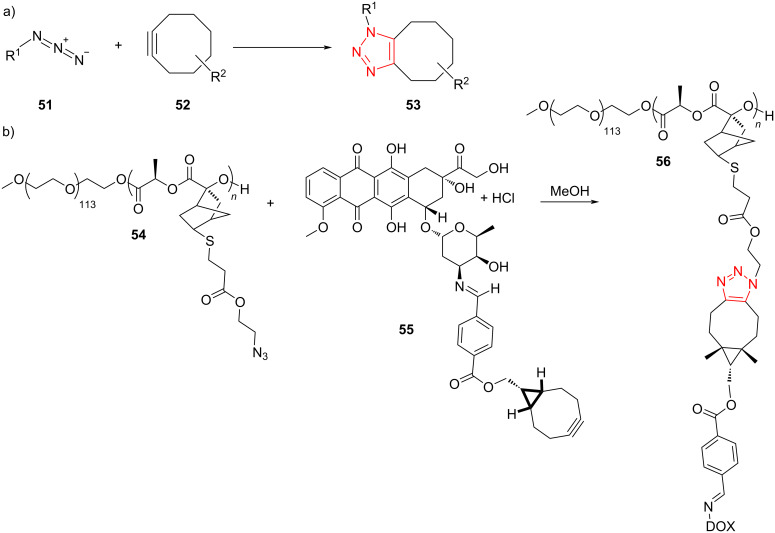
a) A general route for SPAAC in polymer chemistry and b) synthesis of a novel pH-sensitive polymeric prodrug **56** via SPAAC Click chemistry.

Some research groups have utilized strain-promoted azide–alkyne cycloaddition reactions (SPAAC) on side chains to afford polymer-based prodrugs. Generally, a range of key strained cyclooctyne derivatives **52** could be reacted with aliphatic azides **51** via this strategy to give fully decorated triazoles **53** (Scheme 18a). A research group reported a novel polymer **56** functionalized by doxorubicin (DOX). To produce this prodrug, cyclooctyne-derivatized doxorubicin **55** was grafted on an azide-functionalized polymer **54**. In the final step of the construction of this prodrug, the reaction between **54** and **55** was performed in anhydrous methanol under an argon atmosphere to construct **56**. The study on the in vitro drug release showed enhanced drug release at an acidic pH value in comparison to a neutral pH value. Importantly, the amount of DOX required for MCF-7 cells was decreased after the support of DOX molecules on the polymer prodrug, which resulted in reduced side effects (Scheme 18b) [48–49].

### Cu-catalyzed synthesis of fully decorated triazoles

**Scheme 19:**
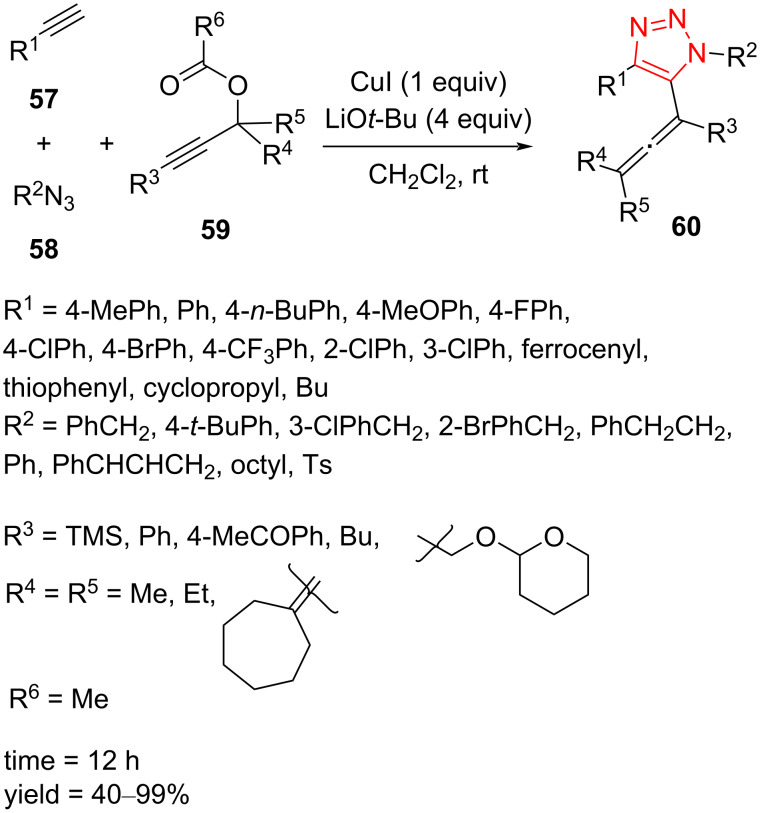
Synthesis of 5-allenyl-1,2,3-triazoles **60** by the treatment of alkynes **57**, azides **58**, and propargylic carbonates **59**.

The Cu-catalyzed regioselective cyclization of alkynes **57** and azides **58**, followed by coupling with propargylic carbonates **59**, afforded the corresponding 5-allenyl-1,2,3-triazoles **60** in moderate to excellent yield. The reaction was tolerant to the presence of a range of aryl-, alkyl-, and heteroaryl-substituted alkynes as well as aryl and alkyl azides. Subsequently, the scope of propargylic carbonates was further explored, altogether leading to a moderate to high yield of the products (Scheme 19) [50].

**Scheme 20:**
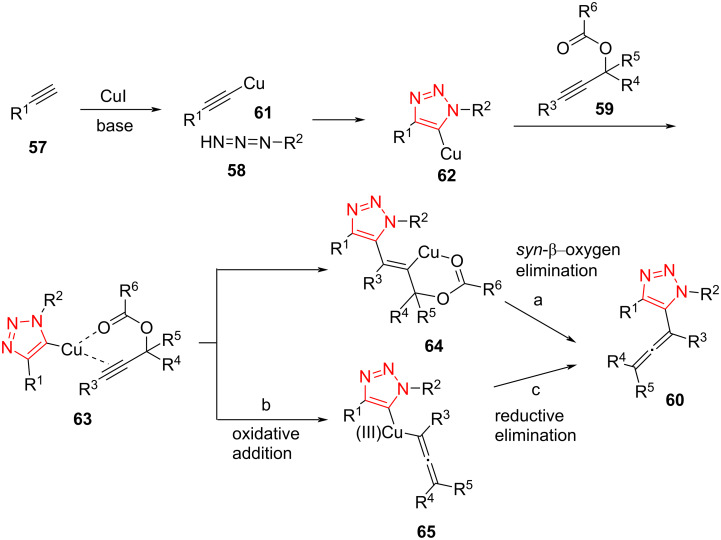
A reasonable mechanism for the synthesis of 5-allenyl-1,2,3-triazoles **60** by the treatment of alkynes **57**, azides **58**, and propargylic carbonates **59**.

A reasonable mechanism for this cyclization/coupling reaction involves the generation of a 5-copper(I)-substituted triazolide intermediate **62**, which coordinates with propargyl carbonate **59**. Further insertion of the C–C triple bond of an alkyne into the Cu–triazole bond gives **64**, which then undergoes *syn*-β-oxygen elimination to provide the target product. On the other hand, an oxidative addition and reductive elimination sequence can also generate the target product (Scheme 20) [50].

**Scheme 21:**
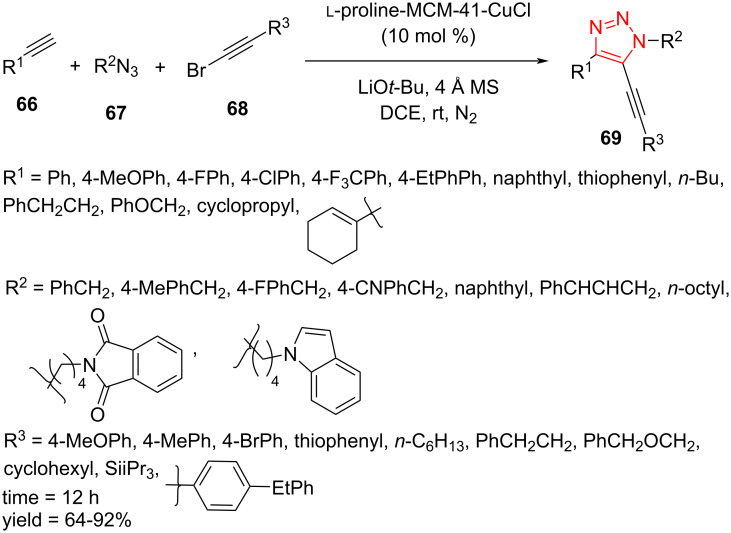
Synthesis of 5‐alkynyl-1,2,3-triazoles **69**.

An immobilized copper complex has been found to be efficient for a practical pathway to 5‐alkynyl-1,2,3-triazoles **69** from organic azides **67**, alkynes **66**, and 1-bromoalkynes **68**. To test the scope of this reaction, a number of alkyl- and (hetero)aryl-substituted alkynes as well as (alkyl)aryl azides have been used in this reaction, affording 5-alkynyl-1,2,3-triazoles **69** in good to high yield. Moreover, heteroaryl-substituted 1-bromoalkyne, iPr_3_Si-substituted 1-bromoalkyne, and a number of alkyl-substituted 1-bromoalkynes were treated in this process to provide the corresponding products with good to high yield (Scheme 21) [51]. It is noteworthy that the immobilized copper complex has been synthesized in some steps. Initially, MCM-41 was reacted with 2-(4-(chloromethyl)phenyl)ethyltrimethoxysilane in toluene at 110 °C for 24 h. The resulting material was then treated with Me_3_SiCl in toluene at mild temperature to afford the chloromethyl-modified MCM-41 (ClCH_2_-MCM-41). In the next step, ʟ-proline-modified MCM-41 (ʟ-proline-MCM-41) was produced by the treatment of ClCH_2_-MCM-41 with *N*-Boc-*trans*-4-hydroxy-ʟ-proline in THF in the presence of NaH as base, which was then deprotected using TFA in CH_2_Cl_2_. In the last step, the immobilized copper complex (ʟ-proline-MCM-41-CuCl) was obtained by the reaction of ʟ-proline-MCM-41 with CuCl in acetone at mild temperature (Scheme 21) [51].

**Scheme 22:**
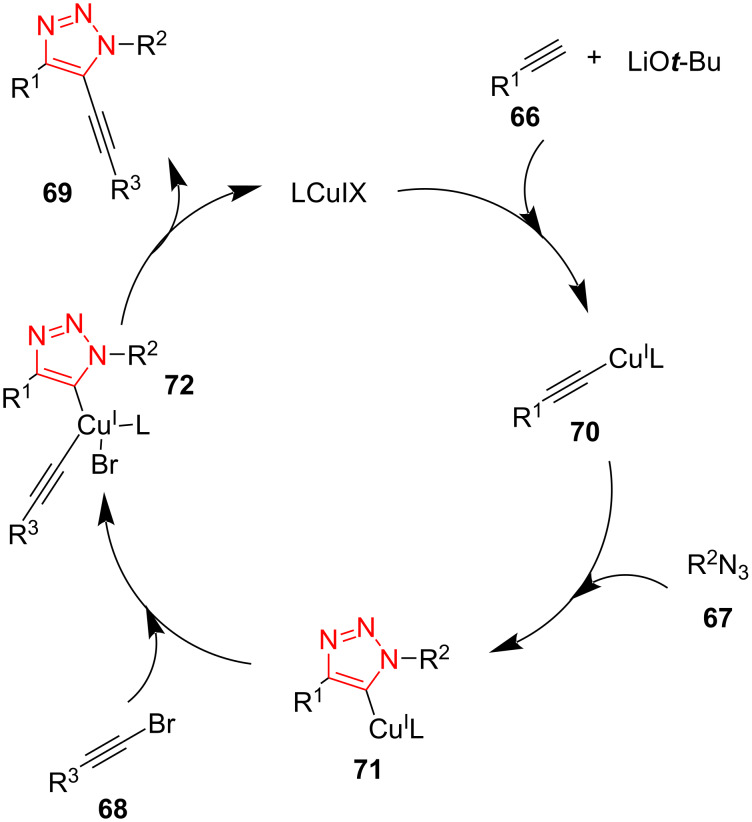
A reasonable mechanism for the synthesis of 5‐alkynyl-1,2,3-triazoles **69**.

A possible mechanism for this reaction is shown in Scheme 22. Initially, copper(I)-substituted acetylide intermediate **70** is produced via the reaction of copper catalyst with the corresponding acetylide by using LiO*t*-Bu. Further intermolecular [3 + 2]-cycloadditions of azide **67** with intermediate **70** affords a 5-copper(I)-substituted triazolide intermediate **71**. The oxidative addition of 1-bromoalkyne **68** forms an alkyne–Cu(III)Br–triazole complex intermediate **72**. Finally, a reductive elimination takes place to give the corresponding final product and the catalyst for the next run [51].

**Scheme 23:**
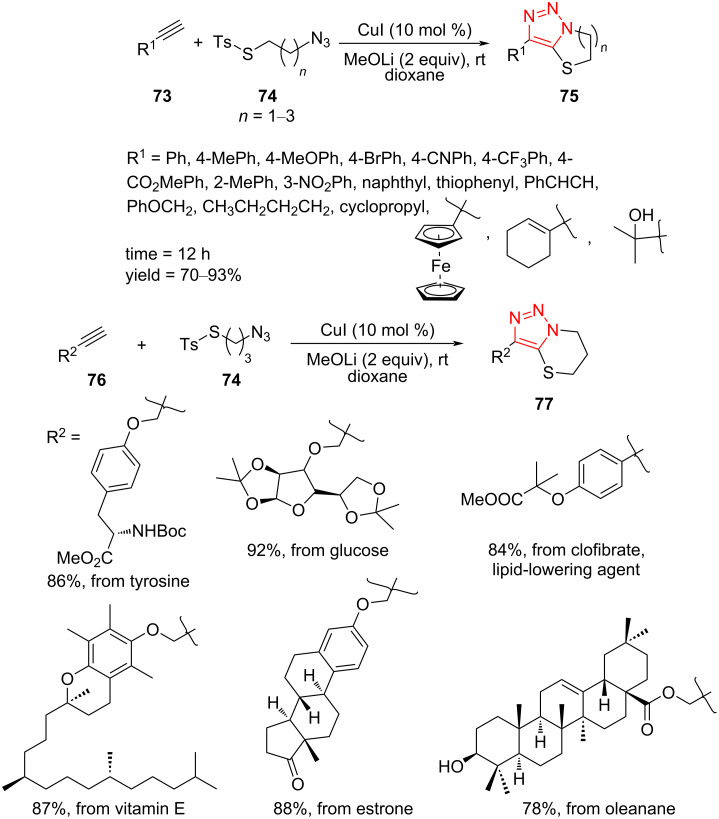
Synthesis of sulfur-cycle-fused 1,2,3-triazoles **75** and **77**.

A tandem Click/intramolecular sulfenylation procedure for the synthesis of sulfur-cycle-fused 1,2,3-triazoles **75** and **77** was described by Xu et al. The reaction was performed through a [3 + 2]-cycloaddition between alkynes **73** and **76** and alkylthiotosyl azides **74** using MeOLi in the presence of a copper(I) catalyst in dioxane at room temperature. This Click/intramolecular sulfenylation reaction displayed an extensive scope, complete regioselectivity, and good to high yield of products, allowing the construction of a number of medium- and large-sized triazole-fused heterocycles **75** and **77**. Many aliphatic, aromatic, thiophenyl-, and ferrocenyl-substituted acetylenes were found to give excellent results in the reaction with aliphatic tosylthio azides **74** with different chain length (*n* = 1–3). For azides with *n* = 3, the reaction was performed using alkynes bearing rather complex R^2^ substituents, such as tyrosine, glucose, clofibrate, vitamin E, estrone, and oleanane triterpenes, which tolerated the reaction conditions and afforded the corresponding triazoles **77** fused with a six-membered sulfur-containing cycle (Scheme 23) [52].

**Scheme 24:**
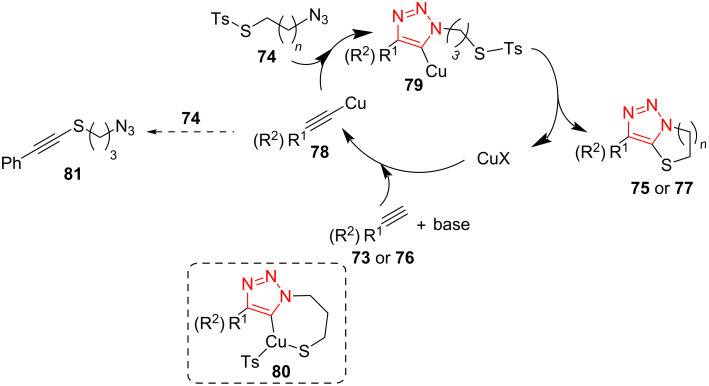
A reasonable mechanism for the synthesis of sulfur-cycle-fused 1,2,3‐triazoles **75** and **77**.

Some reactions were performed to screen the reaction mechanism in detail. To find out if the reaction proceeds through a radical pathway, tetramethylpiperidine-1-oxyl (TEMPO) was added to the reaction. The corresponding product was obtained in high yield, which displayed that a radical pathway may not be involved. The compound **80** also failed to give the corresponding product under standard conditions, which exhibits that **80** is not an intermediate in this reaction. On the other hand, the 1,4-disubstituted 1,2,3-triazole did not convert to the corresponding product under the standard conditions, denoting that the reaction did not include the sequence of CuAAC followed by C–H activation. According to these facts, the reaction mechanism may be as below. First, the copper(I)-substituted acetylide **78** was generated via the reaction of the copper source and the alkyne **73** or **76** using a base. This subsequently cyclized with the azide **73** to afford the triazole cuprate intermediate **79**. Finally, the resulting intermediate proceeds through an oxidative addition and reductive elimination sequence to give the final product, followed by the next cycle (Scheme 24) [52].

**Scheme 25:**
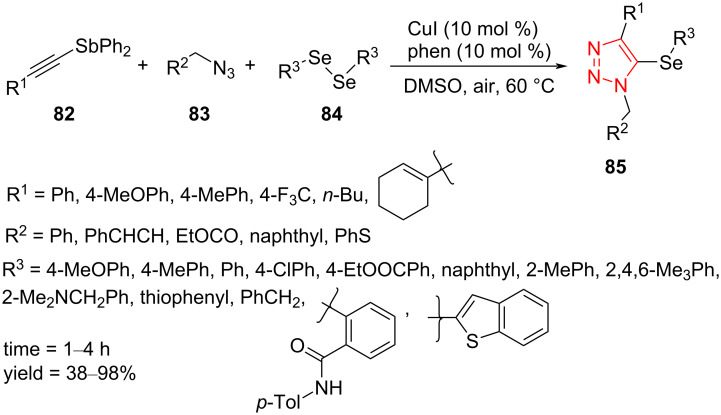
Synthesis of 5-selanyltriazoles **85** from the reaction of ethynylstibanes **82**, organic azides **83**, and diaryl diselenides **84**.

A one-pot and multicomponent protocol for the synthesis of 5-selanyltriazoles **85** from the reaction of ethynylstibanes **82**, organic azides **83**, and diaryl diselenides **84** using catalytic amounts of CuI and 1,10-phenanthroline in DMSO at 60 °C under air with good to high yield was presented by Yasuike et al. (Scheme 25). To find the scope of this strategy, diverse ethynylstibanes **82** containing substituents including aryl, vinyl, and alkyl were reacted with organic azides **83** and diaryl diselenides **84** to form desired products. In addition, a variety of organic azides **83** containing substituents such as cinnamyl, (ethoxycarbonyl)methyl, 1-naphthalenemethyl, and (phenylthio)methyl were cyclized with ethynylstibanes **82** and diaryl diselenides **84** to afford the desired triazole products. The reaction between ethynylstibane, organic azide, and a range of diaryl diselenides **84** including sterically hindered *o*-substituted and heterocyclic derivatives efficiently proceeded to give the desired 5-selanyl-1,2,3-triazoles **85** [53].

**Scheme 26:**
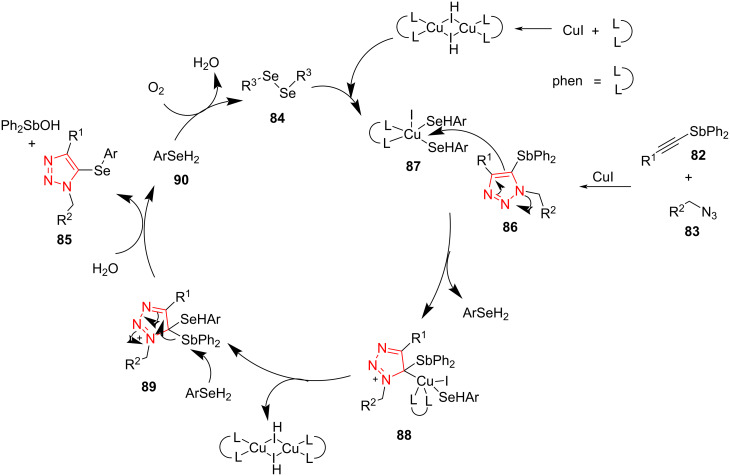
A mechanism for the synthesis of 5-selanyltriazoles **85** from the reaction of ethynylstibanes **82**, organic azides **83**, and diaryl diselenides **84**.

A possible mechanism was suggested and is displayed in Scheme 26. First, the Cu(I)-catalyzed cyclization of ethynylstibane **82** and azide **83** gives the intermediate **86**, which attacks the Cu(III) intermediate **87**, formed via the reaction between diaryl diselenide **84** and the bimetallic complex [L_2_Cu]_2_I_2_. Next, the produced intermediate **88** undergoes a reductive elimination to achieve intermediate **89** with regeneration of the Cu complex [L_2_Cu]_2_I_2_. Then, nucleophilic attack of the aryl selenide anion to the antimony atom takes place to give 5-selanyltriazole **85** and Ph_2_Sb–SeAr. Ph_2_Sb–SeAr is hydrolyzed in aqueous medium to give selenol **90** and Ph_2_SbOH. Selenol **90** is oxidized in air and transformed into diselenide **84**. Therefore, only 0.5 equiv of diaryl diselenide were necessary for the reaction as both of the two selanyl groups are used [53].

**Scheme 27:**
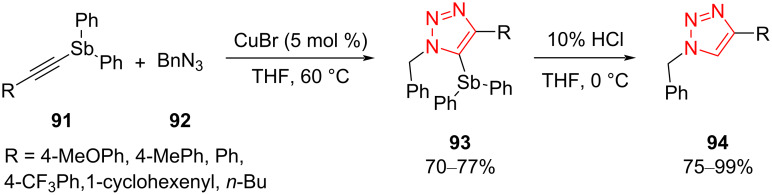
Synthesis of trisubstituted triazoles containing an Sb substituent at position C5 in **93** and 5-unsubstituted triazoles **94**.

Trisubstituted triazoles **93** containing an Sb substituent at position C5 were prepared via a Cu-catalyzed [3 + 2]-cycloaddition reaction between several ethynylstibanes **91** and benzyl azide **92** using CuBr under air. The reaction proceeded to afford 5-stibanotriazoles **93** in good yield (Scheme 27) [54]. The 5-stibanotriazoles **93** were used as intermediates for the synthesis of 5-unsubstituted triazoles **94** in the presence of HCl (10%) in THF at 0 °C. The antitumor activity of 5-stibanotriazoles **93** as well as 5-unsubstituted triazoles **94** was explored using some tumor cell lines. The results exhibited that 5-stibanotriazoles **93** exerted a superior antitumor activity. Contrariwise, 5-unsubstituted 1,2,3-triazoles showed very low antitumor activity in comparison to 5-stibanotriazoles. Since 5-unsubstituted 1,2,3-triazoles did not display good antitumor activity in comparison to 5-stibanotriazoles, this reinforces the importance of synthesizing 5-stibanotriazoles [54].

**Scheme 28:**
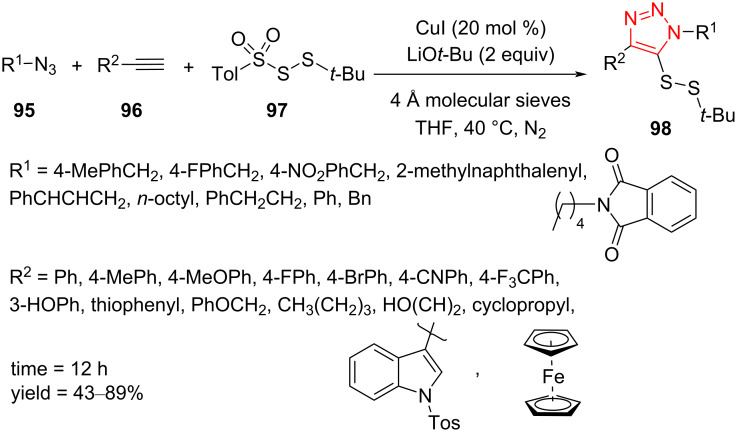
Synthesis of asymmetric triazole disulﬁdes **98** from disulfide-containing *tert*-butyltosyl disulfide **97**, alkynes **96**, and azides **95**.

A similar reaction for the regioselective synthesis of triazole disulﬁdes **98** through a copper(I)-catalyzed cycloaddition of azides **95**, alkynes **96**, and *tert*-butyl tosyl disulfide (**97**) was reported by Xu et al. The reaction has been achieved using a catalytic amount of CuI, LiO*t*-Bu, and 4 Å molecular sieves in THF as solvent at 40 °C under N_2_ atmosphere for 12 h and proceeds via a multicomponent CuAAC/persulfuration sequence. The strategy features a wide substrate scope, and a wide variety of aliphatic moieties, (hetero)aromatic units with electron-donating and electron-withdrawing groups, respectively, and ferrocenyl-substituted terminal alkynes was screened. Diverse triazoles disulﬁdes **98** were achieved in good to high yield. Several aliphatic and aromatic azides were screened, producing a moderate to high yield of the desired products. Moreover, alkynes based on complex molecules, such as glucose, tyrosine, clofibrate, estrone, oleanane triterpenes, and vitamin E tolerated the reaction conditions and afforded the corresponding triazolo disulﬁdes (Scheme 28) [55].

**Scheme 29:**
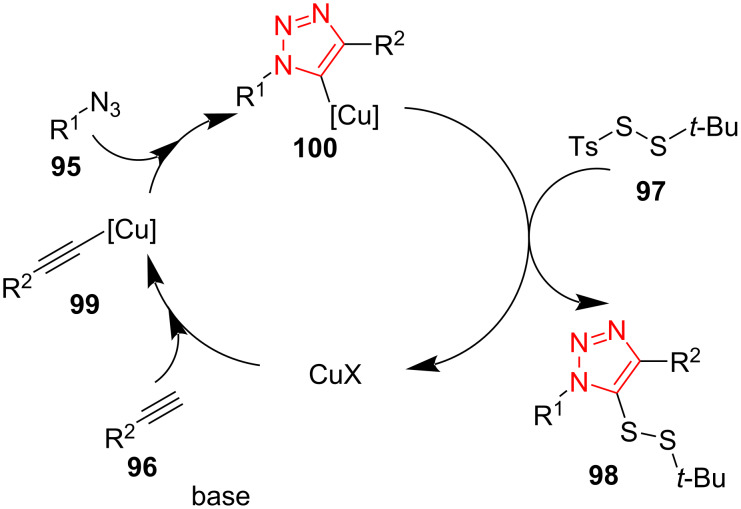
A mechanism for the synthesis of asymmetric triazole disulﬁdes **98** from disulfide-containing *tert*-butyl tosyl disulfide (**97**), alkynes **96**, and azides **95**.

A probable mechanism for this transformation was proposed as illustrated in Scheme 29. The cycloaddition reaction between copper(I) acetylide **99** and organic azide **95** occurs to obtain the triazole cuprate intermediate **100**, which reacts as a nucleophile with the disulfide electrophile **97** to produce the corresponding triazole disulfide **98** [55].

**Scheme 30:**
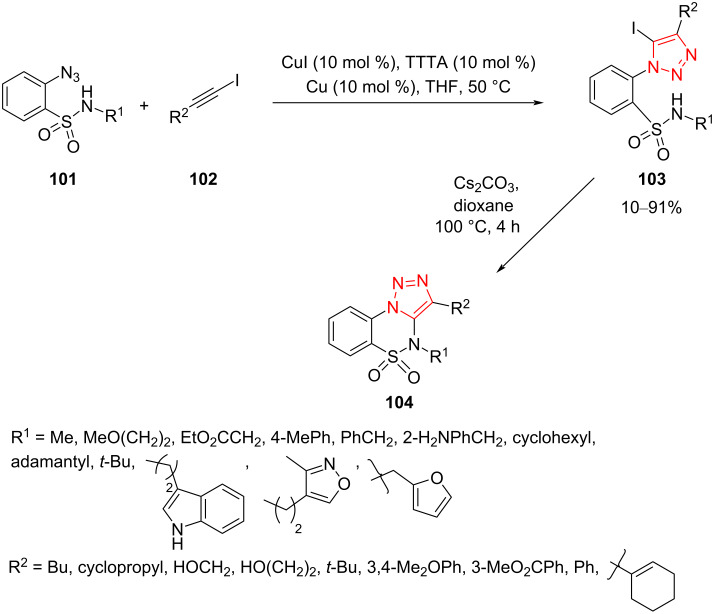
Synthesis of triazole-fused sultams **104**.

A convenient route to triazole-fused sultams **104** was reported by Latyshev et al. It comprises a modified Cu^I^AAC cycloaddition to produce intermediate sulfonamide-tethered 5-iodo-1,2,3-triazoles **103**, followed by a base-mediated cyclization under catalyst-free conditions. A series of azidobenzenesulfonamides **101** was first synthesized from commercially available orthanilic acid by a three-step procedure and then submitted to cycloaddition with different alkynes **102** using CuI and Cu as copper source, tris([1-(*tert*-butyl)-1*H*-1,2,3-triazol-4-yl]methyl)amine (TTTA) as ligand, and THF as solvent at 50 °C. The obtained sulfonamide-tethered 5-iodo-1,2,3-triazoles **103** were then cyclized upon heating in the presence of Cs_2_CO_3_ to give triazole-fused sultams **104**. A good to excellent yield of sultam derivatives **104** containing aryl and alkyl substituents on the triazole ring was achieved under optimized conditions. The reaction displayed extensive diversity and excellent functional group tolerance It should be noted once again that Cu-catalyzed triazoles were obtained in good to excellent yield by using 10% CuI and 10% Cu, which were subsequently converted to fused sultams (Scheme 30) [56].

**Scheme 31:**
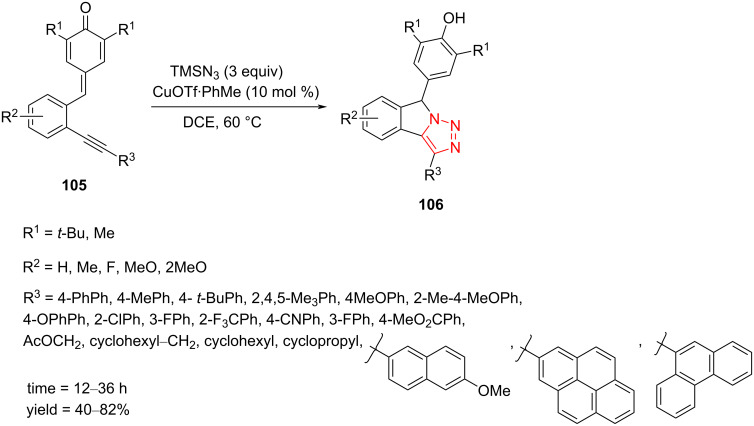
Synthesis of 1,2,3-triazole-fused tricyclic heterocycles **106**.

Anand et al. reported a Cu-catalyzed one-pot method for the preparation of 1,2,3-triazole-fused tricyclic heterocycles **106** via an intramolecular [3 + 2]-annulation. This strategy includes the 1,6-conjugate addition of Me_3_SiN_3_ to *o-*alkynylated *p*-quinone methides **105**, followed by an intramolecular Click annulation to provide rapid access to a wide range of desired 1,2,3-triazole-fused isoindolines **108** with a reasonable yield. The reaction was carried out well by using *o*-alkynylated *p*-quinone methides **105** and TMSN_3_ in the presence of a catalytic amount of CuOTf⋅PhMe in DCE at 60 °C. A series of *o*-alkynylated *p*-quinone methides containing electron-donating, electron-withdrawing, halogen, and alkyl groups was treated with Me_3_SiN_3_ under the optimized reaction conditions and produced the desired fused 1,2,3-triazole derivatives (Scheme 31) [57].

**Scheme 32:**
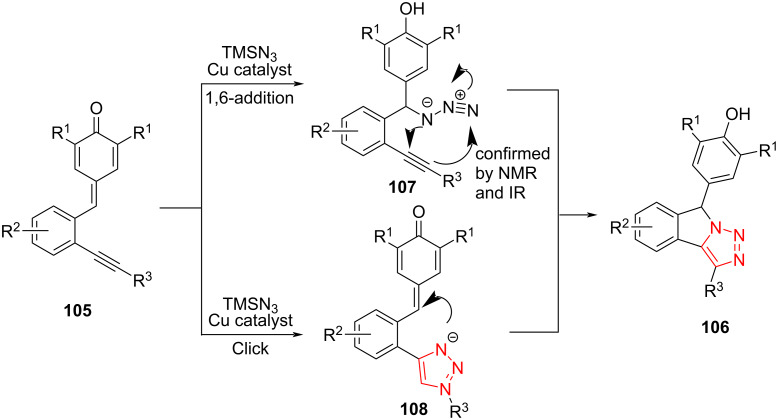
A reasonable mechanism for the synthesis of 1,2,3-triazole-fused tricyclic heterocycles **106**.

Two mechanisms for this transformation can be assumed. First, the *o*-alkynylated *p*-quinone methide **105** undergoes a 1,6-conjugate addition with Me_3_SiN_3_ to give the intermediate **111**, followed by the intramolecular Click annulation to afford the final product. A further possibility may be the creation of the 1,2,3-triazole intermediate **107**, followed by intramolecular 1,6-conjugate addition to afford the final product. To find the exact mechanism, the reaction was performed in CDCl_3_ as solvent in an NMR tube with a catalytic amount of AgSbF_6_ and then, the reaction mixture was analyzed using ^1^H NMR spectroscopy. The ^1^H NMR spectrum displayed two peaks at 6.28 and 5.19 ppm related to the benzylic and the phenolic OH protons, respectively, of the intermediate **106** (Scheme 32) [57].

**Scheme 33:**
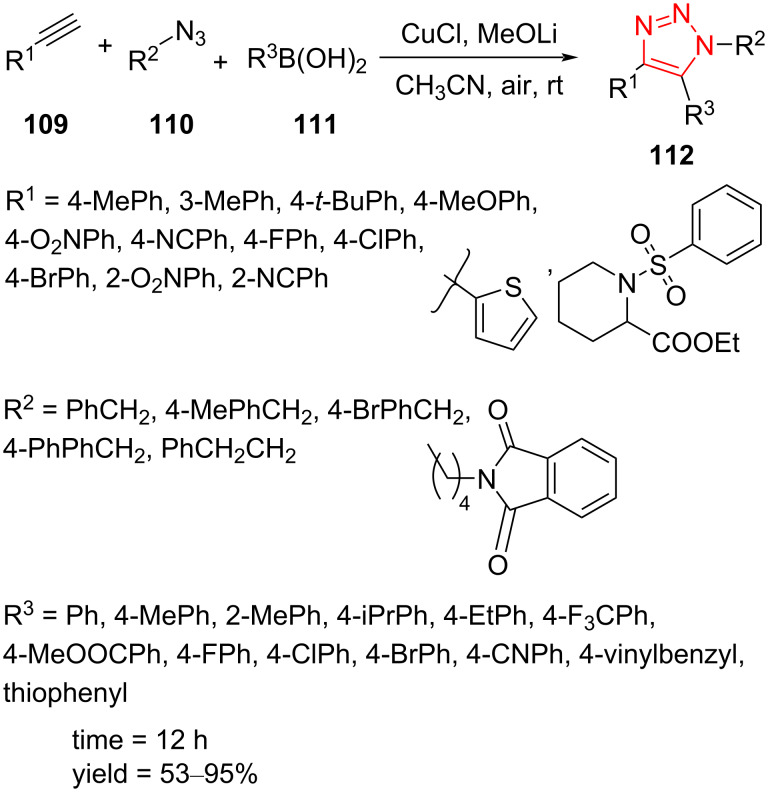
Synthesis of 5-aryl-substituted 1,2,3-triazole derivatives **112**.

Preparation of 5-aryl-substituted 1,2,3-triazole derivatives **112** through a Cu-catalyzed reaction of alkynes **109**, azides **110**, and (hetero)arylboronic acid **111** was reported. The use of a catalytic amount of CuCl and MeOLi as base in CH_3_CN as solvent was recognized as the optimized conditions. This strategy provided a new procedure for the one-pot synthesis of fully decorated 1,2,3-triazole derivatives **112** in good to excellent yield at room temperature. First, several nonactivated arylalkynes containing electron-rich and electron-deficient groups as well as thiophene derivatives afforded the desired 5-arylated triazole derivatives. Then, the diversity of arylboronic acids was explored. The arylboronic acids containing some electron-rich, electron-deficient, and halogen moieties were good partners in these reactions as well, affording the 5-arylated triazoles with good to excellent yield (Scheme 33) [58].

**Scheme 34:**
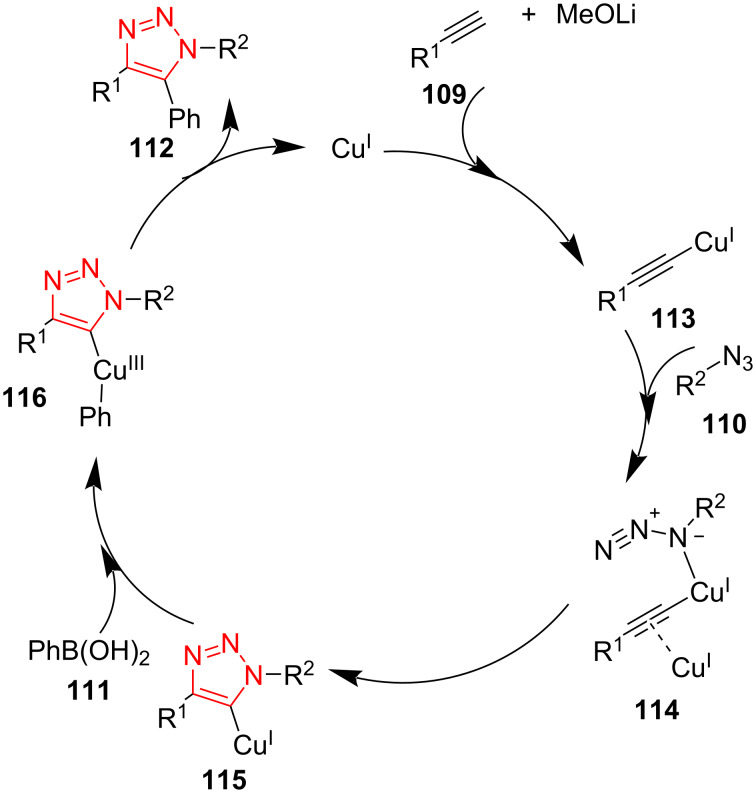
A reasonable mechanism for the synthesis of 5-aryl-substituted 1,2,3-triazole derivatives **112**.

A probable mechanism for this transformation is illustrated in Scheme 34. A copper acetylide intermediate **113** is first formed, which trough the CuAAC path generates the C5-cuprate triazole intermediate **115**. The oxidative addition of arylboronic acid to the copper center forms the intermediate **116**. The reductive elimination occurs to give the corresponding triazole **112** and to reform the copper catalyst for the next run [58].

### Postfunctionalization by Pd- or Pd/Cu-catalyzed synthesis of fully decorated triazoles

**Scheme 35:**
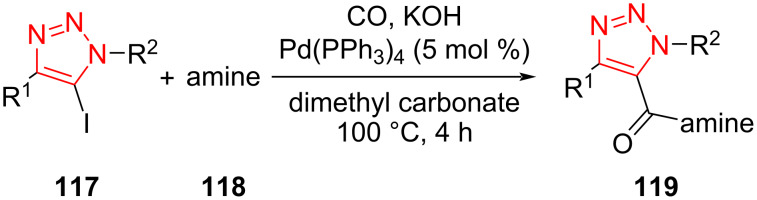
Synthesis of 1,4,5-trisubstituted 1,2,3-triazole-5-carboxamides **119**.

A palladium-catalyzed aminocarbonylation reaction of 5-iodo-1,2,3-triazoles to give 1,4,5-trisubstituted 1,2,3-triazole-5-carboxamides **119** was introduced by Schwab et al. in 2019. The 5-iodo-1,2,3-triazole **117**, amine **118**, and CO were reacted in the presence of a catalytic amount of Pd(PPh_3_)_4_ as well as KOH in (CH_3_O)_2_CO as solvent to obtain 1,2,3-triazole-5-carboxamides **119**. The reaction with several aliphatic primary amines led almost exclusively to the corresponding products. However, no product was obtained using aliphatic secondary (aromatic) amines. In the next step, the effect of R^1^ and R^2^ groups on the triazole ring was also screened. These groups could be aliphatic or aromatic scaffolds. In almost all cases, the desired triazole compounds were formed in good to high yield. The short reaction time, high yield, the use of dimethyl carbonate as a sustainable solvent, the use of an efficient alternative source of carbon monoxide, and avoiding a pressurized cylinder are some benefits of this protocol (Scheme 35) [59].

**Scheme 36:**
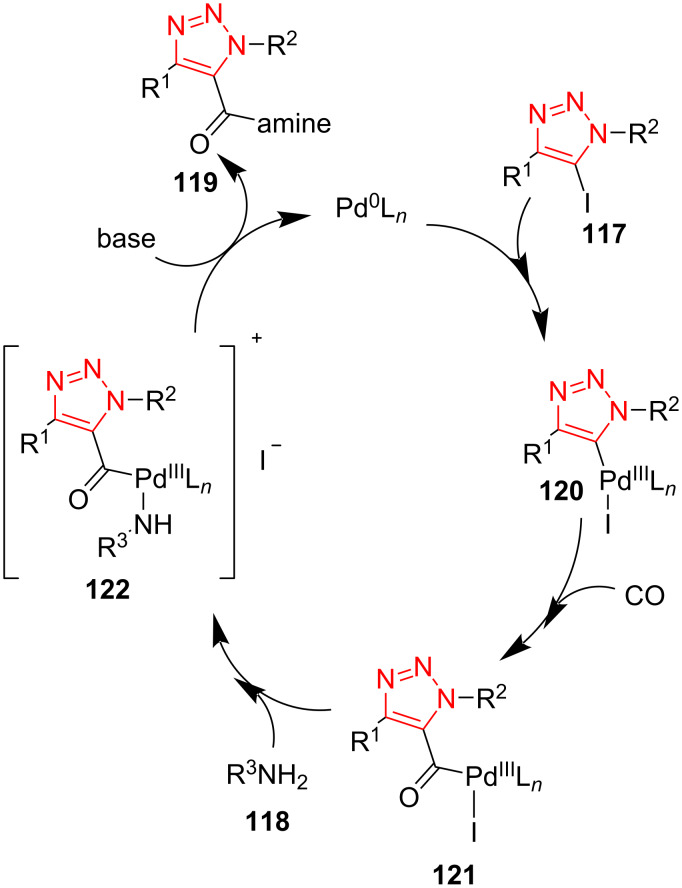
A probable mechanism for the synthesis of 1,4,5-trisubstituted 1,2,3-triazole-5-carboxamides **119**.

A feasible mechanism is illustrated for the synthesis of 1,2,3-triazole-5-carboxamides. The reaction includes oxidative addition of 5-iodo-1,2,3-triazole **117** to a Pd center to achieve the intermediate **120**. The intermediate **121** is formed via coordination and insertion of CO to intermediate **120**. Then, the intermediate **122** is obtained via a nucleophilic attack of amine **118**. Finally, a reductive elimination occurs in the presence of base to achieve the desired product **119** (Scheme 36) [59].

**Scheme 37:**
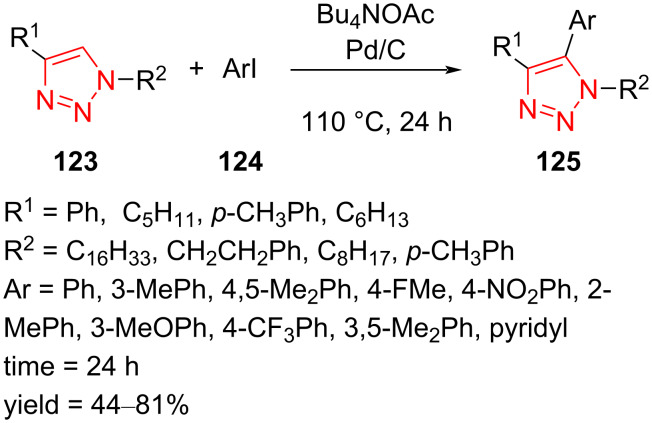
Synthesis of fully decorated triazoles **125** via the Pd/C-catalyzed arylation of disubstituted triazoles **123** with aryl halides **124**.

Direct arylation of disubstituted triazoles **123** with aryl halides **124** using a Pd/C catalyst under solvent-free conditions to give fully decorated triazoles **125** was reported by Farinola et al. Different aryl iodides containing electron-donating and electron-withdrawing groups exhibited good to excellent results in the reaction. Notably, the yield of the Click reaction with 2-iodotoluene decreased due to the steric effects. Moreover, the utilization of an efficient heterogeneous catalyst and insensitivity to air are advantages of this reaction (Scheme 37) [60].

**Scheme 38:**
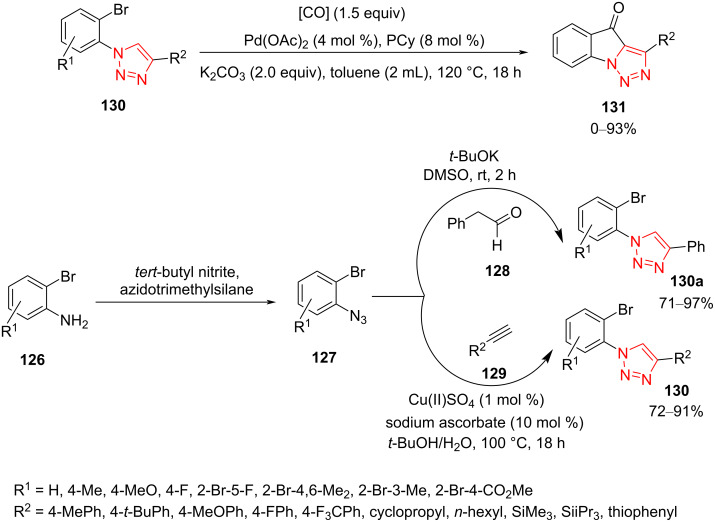
Synthesis of triazolo[1,5-*a*]indolones **131**.

In 2017, De Borggraeve et al. explained a convenient procedure for the preparation of triazolo[1,5-*a*]indolones **131** through an intramolecular cyclization via an unprecedented Pd-catalyzed carbonylative C–H functionalization of 1-(2-bromoaryl)-1,2,3-triazoles **130**. The triazole derivatives were prepared starting from 2-bromoaniline derivatives **126** that were transformed into desired 1-azido-2-bromobenzenes **127** using *t*-BuONO and TMSN_3_. Next, 2-bromophenyl azide derivatives **127** were reacted with phenylacetaldehyde (**128**) or alkynes **129** to afford sterically hindered 1-(2-bromophenyl)-1,2,3-triazole derivatives **130**. The target compounds, triazolo[1,5-*a*]indolones **131**, were then obtained from **130** in high yield using catalytic amounts of Pd(OAc)_2_ and PCy_3_, carbon monoxide, and potassium carbonate and with heating in toluene at 120 °C. The structure of one of the products was proved by NMR analysis and X-ray crystallography. A number of different substituents was introduced to the bromotriazole scaffold, where unsubstituted and electron-rich derivatives gave a good yield, while the presence of electron-withdrawing groups decreased the product yield (Scheme 38) [61].

**Scheme 39:**
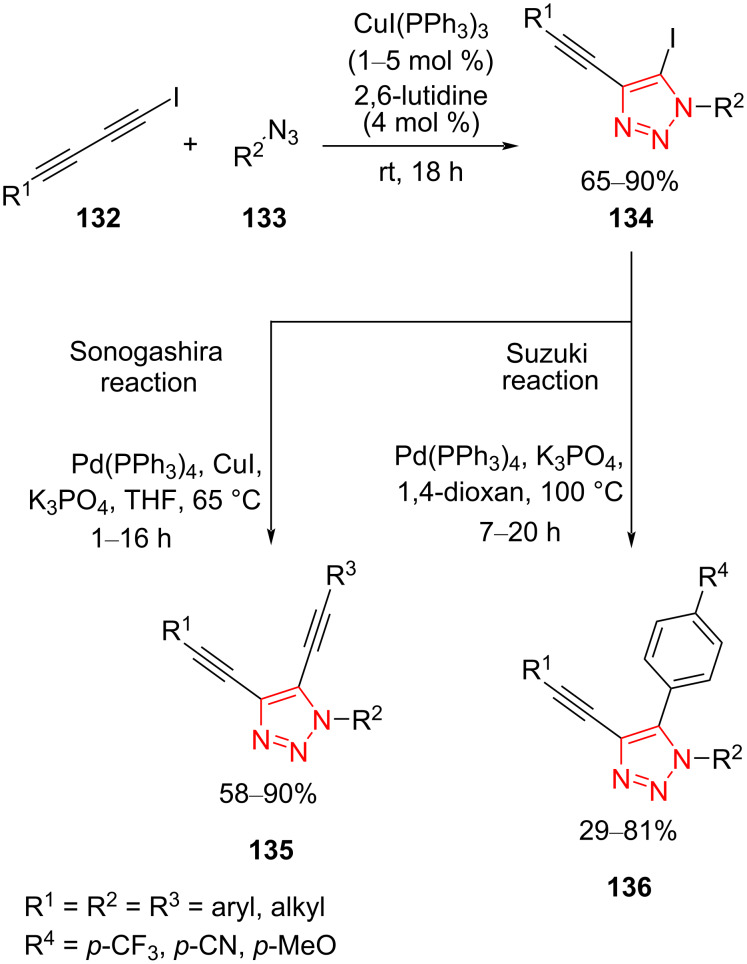
Synthesis of unsymmetrically substituted triazole-fused enediyne systems **135** and 5-aryl-4-ethynyltriazoles **136**.

A number of 4-ethynyl-5-iodo-1,2,3-triazoles **134** have been synthesized through the Cu-catalyzed 1,3-dipolar cycloaddition of iododiacetylenes **132** with organic azides **133** using CuI(PPh_3_)_3_ and 2,6-lutidine as a catalytic system at room temperature. Then, 4-ethynyl-5-iodo-1,2,3-triazoles **134** were used as starting material in the Sonogashira and Suzuki reactions. The Sonogashira−Hagihara cross-coupling led to alkynylation at position 5 of the triazole ring in the presence of CuI and Pd(PPh_3_)_4_ as catalytic system as well as K_3_PO_4_ as base in THF as solvent at 65 °C. However, the Suzuki−Miyaura cross-coupling produced a series of 5-aryl-4-ethynyl triazoles **136** in the presence of Pd(PPh_3_)_4_ as catalyst and K_3_PO_4_ as base in 1,4-dioxane as solvent at 100 °C (Scheme 39) [62].

**Scheme 40:**
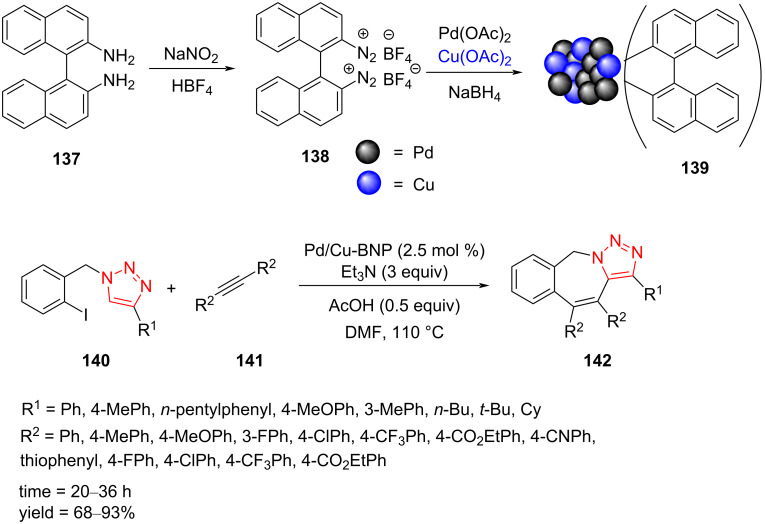
Synthesis of Pd/Cu-BNP **139** and application of **139** in the synthesis of polycyclic triazoles **142**.

A paper by Sekar et al. described the synthesis of polycyclic triazoles **142** through a domino alkyne insertion and C–H bond functionalization reaction sequence with triazole-bearing aryl iodide **140**, catalyzed by alloy-structured Pd/Cu bimetallic nanoparticles stabilized by a binaphthyl moiety, Pd/Cu-BNP **139**. The Pd/Cu-BNP nanoparticles **139** were demonstrated to be an efficient and recyclable catalyst, and triazoles carrying alkyl substituents as well as electron-rich and electron-poor aryl groups afforded the corresponding products **142** in good to excellent yield. A moderate to good yield was achieved when numerous diarylacetylenes **141** substituted by 4-Me, 4-OMe, F, CF_3_, CO_2_Et, and CN were employed in this transformation (Scheme 40). The synthetic route for the Pd/Cu-BNP nanoparticles **139** is also shown in Scheme 40. Pd/Cu-BNP was synthesized by the following procedure as illustrated in Scheme 40. First, 1,1'-binaphthyl-2,2'-diamine (BINAM, **137**) was transformed to [1,1'-binaphthalene]-2,2'-bis(diazonium) tetraﬂuoroborate (**138**) in the presence of HBF_4_ and NaNO_2_ at 0 °C. Next, the nanoparticles were obtained from the reduction of Pd(OAc)_2_ and Cu(OAc)_2_ employing NaBH_4_ in the presence of diazonium salt [63].

**Scheme 41:**
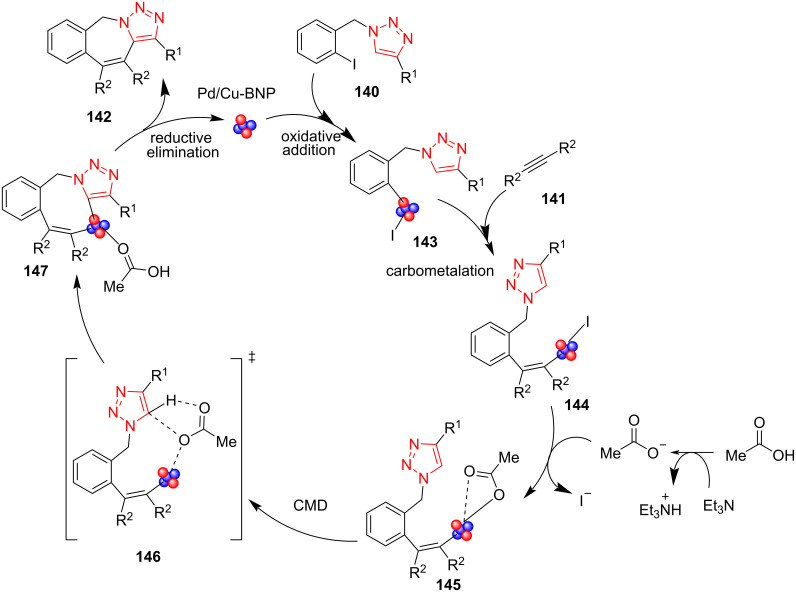
A probable mechanism for the synthesis of polycyclic triazoles **142**.

A possible mechanism for the synthesis of polycyclic triazoles **142** was proposed by Sekar et al. as well [63]. First, the nanocatalyst **139** is inserted in the C–I bond to obtain the intermediate **143**. Then, the insertion of diarylacetylenes **141** forms intermediate **144**. In continuation, the acetate anion formed by reaction of AcOH with Et_3_N undergoes a ligand exchange with iodide to produce the intermediate **145**. Later, carboxylate-ligand-assisted C–H bond activation takes place through a concerted metalation–deprotonation transformation to produce the next intermediate. Finally, the corresponding product **142** is formed by a reductive elimination process, along with the regeneration of the active catalytic species for the subsequent catalytic run (Scheme 41) [63].

**Scheme 42:**
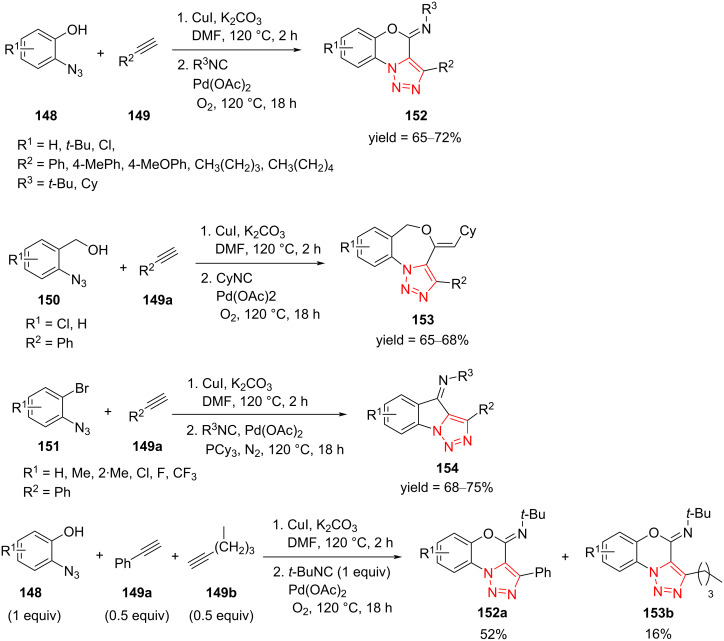
Synthesis of highly functionalized 1,2,3-triazole-fused 5-, 6-, and 7-membered rings **152**–**154**.

Kumar et al. presented a novel protocol for the synthesis of highly functionalized 1,2,3-triazole-fused 5-, 6-, and 7-membered rings **152**–**154** via azide–alkyne cycloaddition, followed by C(sp^2^)−H functionalization of the 1,2,3‐triazole intermediate through an isocyanide insertion. The significant benefits of this strategy were simple substrates, few synthetic steps, and high diversity. A range of *o*‐azidophenols **148** as well as the compounds **150** and **151**, along with alkynes **149** and isocyanides, were employed to survey the diversity of highly functionalized tricyclic triazoles **152**–**154**. It was proved that *o*‐azidophenols **148** react well with alkynes **149** using CuI in DMF at 120 °C and then, the resulting intermediates react with isocyanides using Pd(OAc)_2_ and an O_2_ atmosphere at 120 °C to produce the desired products (Scheme 42). *o*‐Azidophenols **148** were treated with aryl and aliphatic alkyne partners and then with *tert*-butyl and cyclohexyl isocyanide to give 1,2,3‐triazole-fused oxazines **152**. The reaction was extended to 2‐(azidophenyl)methanol species **150**, which were treated with ethynylbenzene and cyclohexyl isocyanide, and in all cases, the corresponding oxazepines **153** were obtained in good yield [63].

The 1-azido-2-bromobenzenes **151** with H, Me, F, Cl, and CF_3_ substituents, respectively, on the phenyl ring were then tested in the reaction with phenylacetylene (**149a**), followed by treatment with *tert*‐butyl isocyanide or cyclohexyl isocyanide under nearly the same conditions to give diverse 1,2,3‐triazole-fused indolylidenes **154**. In this case, it was necessary to use PCy_3_ as a ligand for Pd and N_2_ atmosphere.

Finally, the reactivity of aromatic and aliphatic alkynes was compared performing the reaction of *o*‐azidophenol **148a** (1 equiv), phenylacetylene (**149a**, 0.5 equiv) and aliphatic alkyne **149b** (0.5 equiv) with *tert*‐butyl isocyanide (1.0 equiv) under standard reaction conditions (Scheme 42). It was found that the arylalkyne is much more reactive than the aliphatic alkyne under standard reaction conditions, giving the corresponding condensed triazole **152a** [64].

**Scheme 43:**
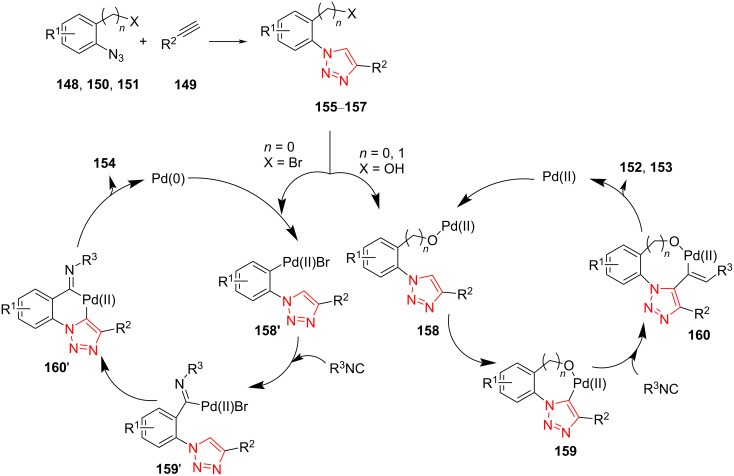
A probable mechanism for the synthesis of highly functionalized 1,2,3-triazole-fused 5-, 6-, and 7-membered rings **152**–**154**, respectively.

A probable mechanism for this transformation is illustrated in Scheme 43. An azide substrate **148**, **150**, or **151** is treated with a nonactivated alkyne **149** using CuI as catalyst and K_2_CO_3_ as base to obtain the 1,4-disubstitued 1,2,3-triazole intermediate **155**–**157**, respectively. The 1,2,3-triazole-based phenol or alcohol coordinates to the Pd center to form complex **158**. Then, the electrophilic palladation of the 1,2,3-triazole ring occurs to achieve Pd(II) intermediate **159**. Isocyanide migrates to **159** to obtain the seven‐ or eight‐membered palladium cycle **160**. The reductive elimination of **160** takes place, which is followed by tautomerization to produce the desired product **152** or **153** and the Pd(0) species, which is then oxidize to Pd(II) in the presence of O_2_. Pd(0) species were inserted into bromo-substituted 1,2,3-triazole **155**–**157**, respectively, to afford further intermediate **158'**. The isocyanide is inserted into intermediate **158'** to achieve intermediate **159'**. A 1,2,3-triazole C–H bond activation occurs using palladium, which subsequently undergoes a reductive elimination process to afford final product **154**. Likewise, the Pd(0) species is reformed for the next cycle [64].

The synthesis of fully decorated 1,2,3-triazoles **162**, **164**, and **166** was reported by Ackermann et al. via intramolecular oxidative C–H arylation of 1,2,3-triazoles **161**, **163**, and **165** using a reusable palladium catalyst in PEG, Pd@PEG, under O_2_ atmosphere. The main advantages of this strategy include i) a versatile methodology for aerobic C–H activation; ii) PEG as green and recyclable reaction medium; and iii) a reusable palladium catalyst complex [65].

**Scheme 44:**
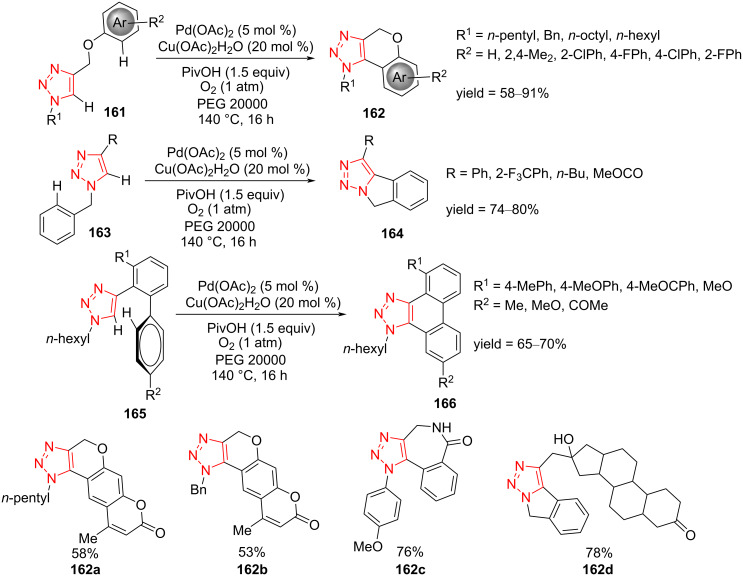
Synthesis of fully functionalized 1,2,3-triazolo-fused chromenes **162**, **164**, and **166** via the intramolecular oxidative C–H arylation of 1,2,3-triazoles **161**, **163**, and **165**.

Fully functionalized 1,2,3-triazolo-fused chromenes **162**, **164**, and **166** were regioselectively prepared in moderate yield. The isoindoline-fused triazoles **164** were achieved using dehydrogenative intramolecular cyclization of 4-substituted *N*-benzyltriazoles **163**. A phenyl, alkyl, and CO_2_Me group, respectively, on the triazole scaffold was tolerated under the reaction conditions to form 4-substituted *N*-benzyltriazoles (Scheme 44). The substituted phenanthro[9,10-*d*]triazoles **166** were also prepared through the cyclodehydrogenative arylation of **165**. It was demonstrated that the Pd and PEG mixture could be reused for further cycles, and only a catalytic amount of the copper source, pivalic acid, and the substrates needed to be added [65]. As shown in Scheme 44, this methodology was extended to the synthesis of potentially bioactive derivatives, such as heterofused coumarins **162a** and **162b**, azepinone-like **162c**, and steroid-based triazolo-fused isoindoline **162d** [65].

### Ru-catalyzed synthesis of fully decorated triazoles

**Scheme 45:**
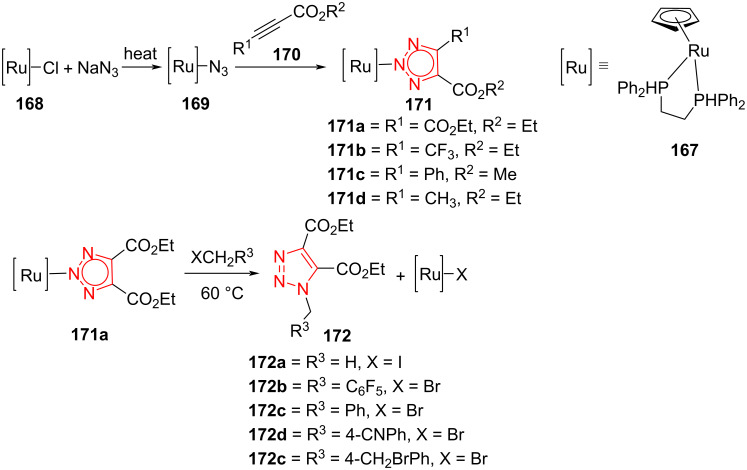
Ru-catalyzed synthesis of fully decorated triazoles **172**.

A series of 1,4,5-trisubstituted triazoles **172** was prepared by Ru-catalyzed [3 + 2]-cycloaddition of ynoate esters **170** with azide to produce metal-bound heterocyclic complexes **171** via an intramolecular cycloaddition reaction. The ynoate esters including diethyl acetylenedicarboxylate, ethyl 4,4,4-trifluoro-2-butynoate, methyl phenylpropiolate, and ethyl 2-butynoate successfully generated the metal-bound heterocyclic complexes. The alkylation reaction of **171a** led to the bond cleavage between ruthenium and nitrogen to produce several 1-alkylated 4,5-bis(ethoxycarbonyl)-1,2,3-triazoles **172** [66]. Herein, the sodium azide initially reacts with [Ru]–Cl **168** to produce [Ru]–N_3_ **169**. Then, the resulting intermediate cyclizes with ynoate ester **170** to form metal-bound heterocyclic complex **171**. This metal-bound heterocyclic complex **171** reacts with alkyl halide to produce final product **172** via a bond cleavage between ruthenium and nitrogen (Scheme 45) [66].

### Cu/Ru-catalyzed synthesis of fully decorated triazoles

**Scheme 46:**
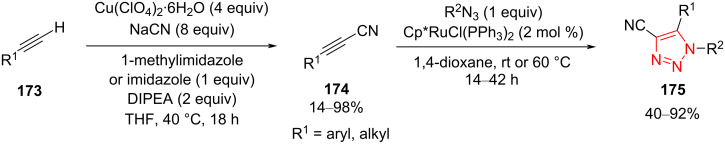
Synthesis of 4-cyano-1,2,3-triazoles **175**.

A novel strategy to transform nonactivated aromatic and aliphatic alkynes **173** to 4-cyano-1,2,3-triazoles **175** was developed by Zhu et al. This reaction involved a two-step sequence. Initially, 1-cyanoalkynes **174** were generated and subsequently, final products were formed through a Ru-catalyzed azide–alkyne cycloaddition process. The current protocol tolerates a range of aromatic and aliphatic azides, affording a diverse range of 4-cyano-1,2,3-triazoles **175** (Scheme 46) [67].

### Ir-catalyzed synthesis of fully decorated triazoles

A series of triazene-functionalized triazoles **182** was synthesized in moderate to high yield through an iridium-catalyzed, directing-group-promoted regioselective [3 + 2]-cycloaddition of alkynes **176** and azides **177**. The optimized conditions for this reaction were found to be Ir(cod)Cl_2_ (2 mol %) and CH_2_Cl_2_ at room temperature. The obtained triazoles **178** were then transformed into the corresponding 5-aminotriazoles **179** by reduction with Raney nickel in MeOH at 60 °C. In addition, the further transformation of 5-aminotriazoles **179** into more complex derivatives, such as **180**–**183**, was explored [68].

**Scheme 47:**
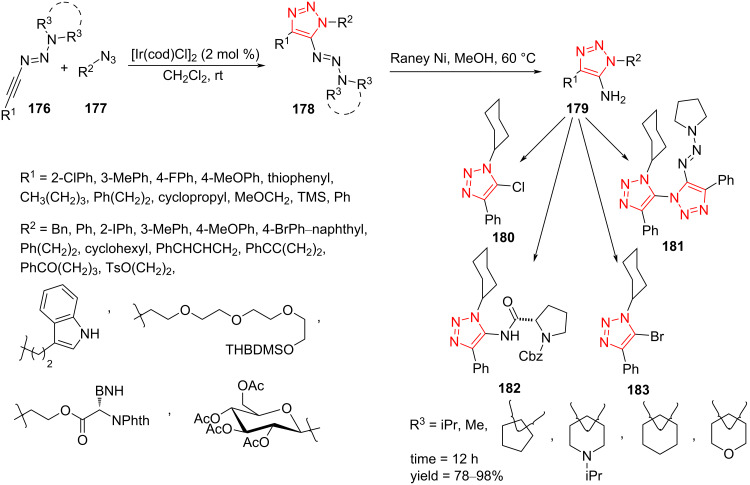
Synthesis of functionalized triazoles from the reaction of 1-alkyltriazenes **176** and azides **177** and synthetic route for functionalization toward diverse triazoles **179**–**183**.

The Huisgen reaction of structurally diverse 1-alkynyltriazenes bearing 2-ClPh, 3-MePh, 4-FPh, and 4-MeOPh, respectively, with azides was explored (Scheme 47). In all cases, a high yield of the triazole derivative was obtained. The products can also be synthesized by alkynes bearing butyl, phenylethyl, cyclopropyl, and methoxymethyl moieties. Moreover, TMS-substituted alkyne was suitable for this Huisgen reaction to achieve the desired triazole product. Next, some triazene scaffolds, such as dimethylamino-, pyrrolidine-, piperidine-, morpholine-, and piperazine-derived triazenes were screened. Aryl azides containing Br, I, Me, and MeO as well as 2-naphthyl azide have been used to give corresponding triazole derivatives efficiently under optimized reaction conditions. Alkyl azides containing functional groups including phenylethyl, indolyl, cyclohexyl, alkenyl, and alkynyl were suitable in this transformation to form the desired products. This method was then extended to ketone, tosyl, and phthalimide groups on alkyl azides to produce the desired triazole derivatives. The ethylene glycol-, phenylalanine-, and glucose-derived azides were also good candidates to furnish the triazole derivatives. This protocol provides access to various substituted triazoles under mild conditions starting from readily available starting materials, showing broad structural diversity (Scheme 47) [68].

**Scheme 48:**
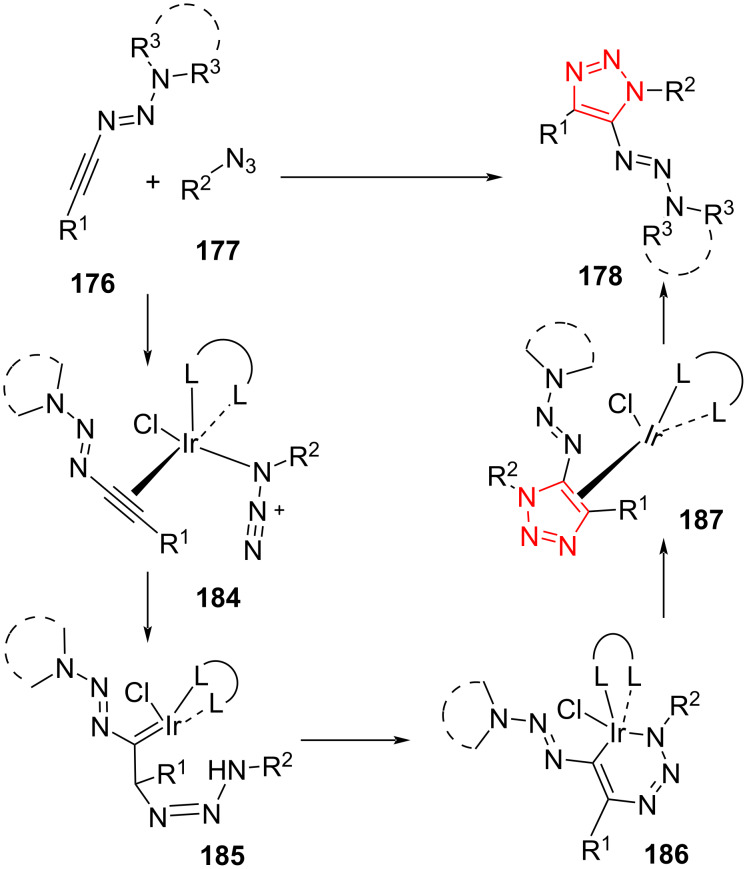
Mechanism for the synthesis of functionalized triazoles from the reaction of 1-alkyltriazenes **176** and azides **177**.

The authors proposed a reasonable mechanism for this process. First, 1-alkyltriazene **176** and azide **177** are coordinated to the iridium center to form intermediate **184**. Subsequently, oxidative coupling of the azide with the β-carbon atom of the 1-alkyltriazene gives the Ir–carbene intermediate **185**. In continuation, intermediate **185** can be transferred to intermediate **186**, leading to the triazole ligand being coordinated to the Ir center in **187**. Finally, the triazole product **178** is released and then, **176** and **177** are coordinated to the Ir center to enable a further catalytic run (Scheme 48) [68].

## Conclusion

Over the last years, the synthesis of highly substituted triazoles has gained extensive interest. This is generally because of the diverse applications of these products in academia, pharmacy, and industry. This overview summarizes the latest advances in the synthesis of fully decorated triazoles. In many cases, the reaction mechanisms of triazole formation have been discussed. The current overview can serve as a base for future advancements in modern protocols for the preparation of new 1,4,5-trisubstituted 1,2,3-triazoles with an extensive range of synthetic, biomedical, and materials applications.
